# Preclinical species gene expression database: Development and meta-analysis

**DOI:** 10.3389/fgene.2022.1078050

**Published:** 2023-01-17

**Authors:** Caitlin Krause, Kinga Suwada, Eric A. G. Blomme, Kenneth Kowalkowski, Michael J. Liguori, Prathap Kumar Mahalingaiah, Scott Mittelstadt, Richard Peterson, Lauren Rendino, Andy Vo, Terry R. Van Vleet

**Affiliations:** ^1^ R & D Data Solutions, AbbVie, North Chicago, IL, United States; ^2^ Development Biological Sciences, AbbVie, North Chicago, IL, United States

**Keywords:** transcript, RNAseq, gene, pre-clinical, tissue, atlas

## Abstract

The evaluation of toxicity in preclinical species is important for identifying potential safety liabilities of experimental medicines. Toxicology studies provide translational insight into potential adverse clinical findings, but data interpretation may be limited due to our understanding of cross-species biological differences. With the recent technological advances in sequencing and analyzing omics data, gene expression data can be used to predict cross species biological differences and improve experimental design and toxicology data interpretation. However, interpreting the translational significance of toxicogenomics analyses can pose a challenge due to the lack of comprehensive preclinical gene expression datasets. In this work, we performed RNA-sequencing across four preclinical species/strains widely used for safety assessment (CD1 mouse, Sprague Dawley rat, Beagle dog, and Cynomolgus monkey) in ∼50 relevant tissues/organs to establish a comprehensive preclinical gene expression body atlas for both males and females. In addition, we performed a meta-analysis across the large dataset to highlight species and tissue differences that may be relevant for drug safety analyses. Further, we made these databases available to the scientific community. This multi-species, tissue-, and sex-specific transcriptomic database should serve as a valuable resource to enable informed safety decision-making not only during drug development, but also in a variety of disciplines that use these preclinical species.

## 1 Introduction

Compound attrition rates remain high for drug companies, despite the significant contributions of animal toxicity studies (Monticello et al., 2017). For example, only ∼10% of Phase 1 candidates reach final approval by the U.S. Food and Drug Administration (FDA), costing well over $1 billion to as much as $4.5 billion (Schlander et al., 2021) U.S. dollars on average to produce a single marketed drug (Munos, 2009; Hay et al., 2014; Blomme and Will, 2016). Lack of efficacy is the greatest cause of drug attrition accounting for ∼57% of these failures (Hwang et al., 2016). However, safety is also a significant cause of drug failures reportedly accounting for 35% and 28% of drug failures from Phase 1 and from Phase 2 to submission, respectively (Arrowsmith and Miller, 2013).

The absence of toxicity in preclinical toxicology species strongly predicts similar outcomes in clinical trials (Monticello et al., 2017). Olson et al. (2000) calculated the sensitivity of the animal to human prediction of 150 drug candidates and concluded that rodents and non-rodents predicted ∼43% and ∼63% of human toxicities, respectively demonstrating the value and limitations of these models. In addition, differences in toxicology/pathology findings in small versus large animal species can make it challenging to understand the human relevance of the findings (Fabre et al., 2020). Species differences in gene expression profile have been linked to differential species sensitivity or resistance to various mechanisms of toxicity (Desforges et al., 2021). Therefore, understanding the translation of specific findings in preclinical toxicology species to human outcomes is critical in making decisions to progress new drug candidates based on safety.

Target safety assessments (TSAs) are an important tool in drug discovery for understanding potential toxicities for a given target *a priori* (Hornberg et al., 2014). As a key component of TSAs, a reliable tissue database of mRNA expression is often used to identify organs and tissues in humans where a given target is expressed and to rank levels of expression between tissues. While well populated human expression databases exist in publicly available formats [e.g., Genotype-Tissue Expression (GTEx)] and have been typically employed for TSAs, equivalent databases in preclinical toxicology species continues to be a clear need (Mele et al., 2015).

With a preclinical database, the target of interest can be queried, and its relative levels of expression between tissues can be examined and interpreted in the context of toxicity assessment and for TSA development. These data may help explain/predict possible species or sex-related differences in sensitivity to drugs. For example, the absence of a particular target-related transcript in a given tissue may suggest that this tissue will be less likely associated with an on-target toxic effect. Likewise, target expression in a tissue of one species, but not of another, can provide a mechanistic understanding of species differences in toxicological outcomes. This knowledge can form the basis of designing specialized exploratory toxicology or *in vitro* studies to confirm suspected effects or to collect certain tissues or endpoints earlier in the development process than typical if an effect is suspected. The TSA, with preclinical species gene expression database information, may also provide a basis for understanding the translation of certain toxicities between preclinical species and humans.

The GTEx database is one of the most extensive gene expression databases containing RNAseq data from a wide range of human donors across multiple organ sites (Stanfill and Cao, 2021). This publicly accessible database provides tremendous value to researchers in understanding the levels of mRNA transcripts, as well as non-coding RNAs, across tissues while also providing information on interindividual variability. No comparable dataset is currently publicly available for preclinical species. In this report, we aim to expand the availability of preclinical transcriptomic data by providing our RNAseq data from males and females of multiple preclinical species (available in GEO) that can be used to determine the location and relative levels of RNA sequences between organs and species. These data could further be compared to existing human RNAseq databases (such as GTEx) to help understand the potential for translation of animal findings to humans.

## 2 Materials and methods

### 2.1 In-life studies and tissue collections

**FIGURE 1:**
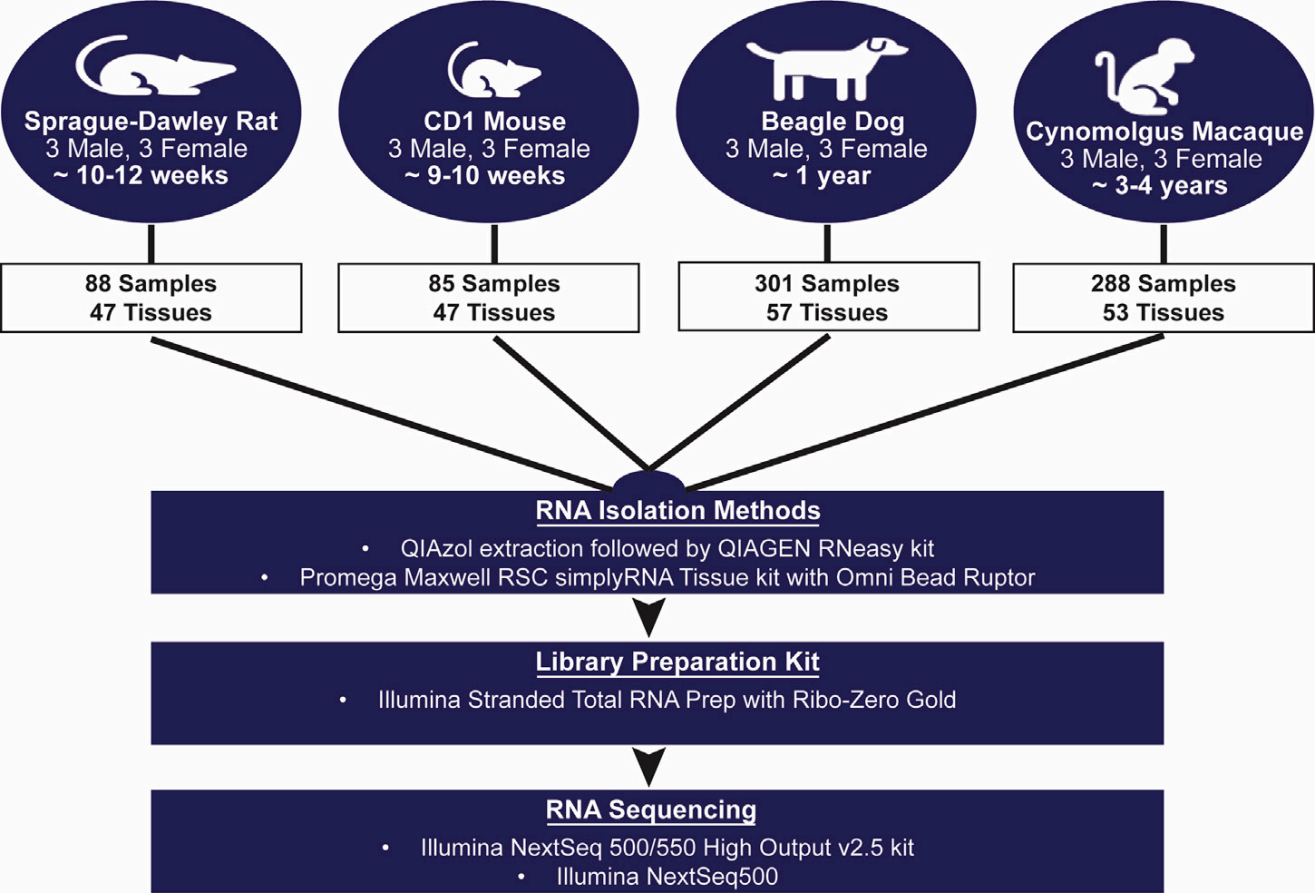
Transcriptomic Body Atlas Study Design. A large number of diverse tissues from mouse, rat, dog, and monkey in males and females were collected separately for RNA isolation and used for RNA-Sequencing and generation of a transcriptomic body atlas. For each species, strains and ages matched those most commonly used in preclinical drug safety studies.

AbbVie is committed to the internationally accepted standard of the 3Rs (Reduction, Refinement, Replacement) and adhering to the highest standards of animal welfare in the company’s research and development programs. Animal studies were approved by AbbVie’s Institutional Animal Care and Use Committee (IACUC) and were conducted in an AAALAC accredited program where veterinary care and oversight was provided to ensure appropriate animal care. Figure 1 shows the overall study design for animal tissue collections and transcript sequencing. Species, strain, age, tissue, and sex were selected to match our internal experimental space for preclinical toxicology studies and toxicogenomic analyses.

Male and female Sprague Dawley rats (*n* = 3/sex), approximately 10–12 weeks old, were euthanized by exsanguination under isoflurane anesthesia and necropsied for tissue collection. Male and female CD-1 mice (*n* = 3/sex), approximately 9–10 weeks old and 20–25 g, were euthanized by exsanguination under carbon dioxide anesthesia and necropsied for tissue collection. Both rats and mice were purchased from Charles River Laboratories, Inc., (Portage, MI, United States) and were then housed (2-3 animals/cage) in plastic, solid bottom hanging cages with bedding, equipped with feeders and an automatic watering system at the AbbVie animal facility. Study animals were fed *ad libitum* the Envigo Teklad Global rodent diet 2014 (Indianapolis, IN, United States) and were fasted overnight prior to the time of necropsy and tissue collection. A total of 47 tissues were collected from each male and female rodent (including 3 to 4 reproductive tissues per sex).

Male and female Beagle dogs (*n* = 3/sex), approximately 1 year old and 5.6–10.7 kg, were purchased from Marshall BioResources (North Rose, NY, United States) housed at the AbbVie animal facility in social housing conditions prior to inclusion in the study and were fed Teklad certified global diet 2025 (Envigo, Madison, WI; *ad libitum*). Dogs were euthanized through intravenous administration of a pharma grade potassium chloride solution or by exsanguination after administration of a barbiturate solution, in compliance with site IACUC, and necropsied for tissue collection. Dogs were fasted overnight (16–18 h) with access to water prior to the time of necropsy. A total of 57 tissues were collected from each male and female dog (including 4 reproductive tissues per sex).

Male and female Cynomolgus monkeys (*n* = 3/sex), approximately 3–4 years old and 2.5–3 kg, were housed at the Charles River Mattawan animal facility prior to inclusion in our study. Lighting, temperature, and humidity were monitored in accordance with institutional SOPs FAC-17 and FAC-52. Monkeys were euthanized through overdose with a barbiturate-based euthanasia agent, propofol, or exsanguination (methods approved by site IACUC) and necropsied for tissue collection. Study animals were fed the Lab Diet Certified Primate Diet #5048 *ad libitum*, were provided tap water *ad libitum via* an automatic water system and were fasted for no more than 1 h prior to necropsy. A total of 53 tissues were collected from each male and female monkey (including 3 reproductive tissues for males and 5 for females).

A complete list of tissues collected in each species is included in Table 1. In all four species, during necropsy, tissues were prioritized for collection according to perceived research value and predicted RNA stability. Upon collection, tissues were transferred into a Whirl-Pak bag and immediately flash-frozen in liquid nitrogen and maintained in freezers at −80°C until RNA isolation. Pancreas was collected first and flash frozen to minimize RNA degradation. A subset of tissues were alternatively rinsed in saline solution and stored in RNALater Stabilization Buffer (Invitrogen, Waltham, MA, United States) and maintained at 4°C until RNA isolation. Tissue storage and preservation conditions were based largely on previous experience with tissues. Upon collection, 100 μl aliquots of whole blood were added to a cryovial containing 1 ml of QIAzol Lysis Reagent (Qiagen, Germantown, MD, United States). Contents of the vial were immediately mixed by inversion and flash frozen in liquid nitrogen until RNA isolations could be performed.

**TABLE 1:** List of Tissues Collected (✓) Across Each Species and Respective Classified Organ Systems. A total of 47–57 tissues were collected for each species. After collection, a portion of tissues was transferred to a Whirl-Pak bag and immediately flash frozen in liquid nitrogen and stored at −80°C.

**Organ Systems**	**Tissues**	**Rat**	**Mouse**	**Dog**	**Monkey**
Respiratory	Lung	✓	✓	✓	✓
	Olfactory Epithelium	-	-	✓	-
	Trachea	-	-	-	✓
Digestive/Excretory	Liver	✓	✓	✓	✓
	Pancreas**	✓	✓	✓	✓
	Tongue	✓	✓	✓	✓
	Ileum*	✓	✓	✓	✓
	Jejunum*	✓	✓	✓	✓
	Duodenum*	✓	✓	✓	✓
	Cecum*	✓	✓	-	-
	Colon^#^	✓	✓	✓	✓
	Stomach^@^	✓	✓	✓	✓
	Esophagus*	✓	✓	-	✓
	Harderian Gland	✓	✓	-	-
	Salivary Glands^$^	✓	✓	✓	✓
	Mouth Mucosa	-	-	✓	✓
	Gallbladder	-	-	✓	✓
	Lacrimal Gland	-	-	✓	✓
Circulatory/Cardiovascular	Aorta	✓	✓	✓	✓
	Heart	✓	✓	✓	✓
	Caudal Vena Cava	✓	✓	-	-
	Femoral Vein	-	^-^	✓	✓
Urinary	Bladder	✓	✓	✓	✓
	Kidney	✓	✓	✓	✓
Integumentary	Skin*	✓	✓	-	✓
	Ear Cartilage	✓	-	-	-
Adipose	White Adipose	✓	✓	✓	✓
	Brown Adipose	✓	✓		
Musculoskeletal	Skeletal Muscle - Gastrocnemius	✓	✓	-	-
	Quadriceps	-	-	✓	✓
	Soleus	-	-	✓	✓
	Gastrocnemius Tendon	-	-	✓	✓
	Synovium	-	-	✓	-
	Physis/Metaphysis	-	-	✓	-
Endocrine	Adrenal Gland	✓	✓	✓	✓
	Pituitary Gland	✓	✓	✓	✓
	Thyroid/Parathyroid	✓	✓	✓	✓
Hemic/Immune	Bone Marrow	✓	✓	✓	✓
	Mesenteric Lymph Node	✓	✓	✓	✓
	Spleen	✓	✓	✓	✓
	Thymus	✓	✓	-	-
	Whole Blood**	✓	✓	✓	-
	Peyer’s Patch*	-	-	✓	✓
Nervous	Cerebellum	✓	✓	✓	✓
	Sciatic Nerve	✓	✓	✓	✓
	Spinal Cord (Cervical)	✓	✓	✓	✓
	Cortex	✓	✓	-	-
	Frontal Cortex	✓	✓	-	-
	Medulla	✓	✓	-	-
	Frontal Lobe	-	-	✓	✓
	Subcortical White Matter	-	-	✓	✓
	Thalamus	-	-	✓	✓
	Caudate Nucleus	-	-	✓	✓
	Hippocampus	-	-	✓	✓
	Dorsal Root Ganglia	-	-	✓	✓
Ocular^*¥*^	Cornea	✓	✓	✓	✓
	Retina	✓	✓	✓	✓
	Uvea	-	-	✓	✓
	Lens	-	-	✓	-
Reproductive	Epididymis	✓	✓	✓	✓
	Prostate	✓	✓	✓	✓
	Testes	✓	✓	✓	✓
	Ovary	✓	✓	✓	✓
	Uterus	✓	✓	✓	✓
	Vagina	✓	✓	✓	✓
	Mammary Tissue	✓	✓	✓	-
	Cervix Uteri	-	✓	✓	✓
	Penis	-	-	✓	-
	Oviduct	-	-	-	✓

^#^Transverse colon in rat and mouse.

^@^Pyloric and fundic stomach collected separately in dog. Pyloric stomach and stomach body collected separately in monkey.

^$^Submandibular and parotic salivary glands collected separately in dog. Submandibular salivary gland in monkey.

^¥^Whole eye (including cornea and retina) was collected in mouse.

* Indicates that tissues were alternatively rinsed in saline solution, stored in RNALater Stabilization Buffer and maintained at 4°C until RNA isolation.

** Indicates that there was a separate protocol used for RNA isolation

### 2.2 Sample collection for clinical pathology and histology

At necropsy, whole blood samples were collected (in rat, dog, and monkey) and used for clinical pathology analysis (standard hematology and clinical chemistry parameters) to confirm that animals included in this study were healthy without any background disease or pathologies that could potentially impact gene expression changes. In mice, due to limited blood volume, samples were not collected for clinical pathology analysis.

In all four species, representative samples were also collected from selected key tissues for histological evaluation to confirm that animals were healthy and without disease. Tissues collected from each species for histology evaluation are listed in Supplementary Table S1. These tissue samples were fixed and then processed for standard paraffin embedding and sectioning. Sections (5 μM) were stained with hematoxylin and eosin and evaluated microscopically by an American College of Veterinary Pathologists-board certified veterinary pathologist.

### 2.3 RNA sample preparation

Total RNA was isolated from collected tissues according to one of several methods, according to our lab’s previous experience with various tissues. Target tissue input was approximately 200–300 mg, with the exception of smaller tissues, particularly in rodents. Flash frozen tissues and RNALater-stored tissues were homogenized in the Omni BeadRuptor (Kennesaw, GA, United States) or by using a Kinematica Polytron homogenizing wand (Malters, Switzerland). Tissue homogenates underwent RNA isolation, DNAse digestion with the Qiagen RNAse-Free DNAse set, and spin-column cleanup with the Qiagen RNeasy Mini kit (Qiagen, Germantown, MD, United States). A portion of the tissues were processed with the Promega Maxwell RSC simplyRNA Tissue Kit (Madison, WI, United States) and the remainder were processed using the Qiagen RNAeasy kit with QIAzol.

RNA quality was assessed using the Agilent 4200 Tapestation System and the Agilent 2100 Bioanalyzer with the Agilent High Sensitivity RNA ScreenTape and Agilent 6000 Nano Kit, respectively (Agilent, Santa Clara, CA, United States). RNA was quantified by the Qubit fluorometer using the RNA BR Assay (ThermoFisher Scientific, Waltham, MA, United States).

### 2.4 RNA sequencing

To ensure that future comparison could be made across species, approximately 50 matching tissues across the four species were selected and classified into broader organ systems based on anatomic structure and/or function (e.g., lymphoid, reproductive) (Figure 1; Table 1). In some individual cases, samples were removed from downstream analyses due to low tissue quality.

For rat and mouse samples, total RNA was pooled in equal mass inputs for each animal to generate a composite sample representative of each tissue. Rodent samples were pooled (within each species), but male and female-derived RNA samples remained separate. Library preparation and sequencing for dog and monkey samples were performed without sample pooling due to greater individual variability in these research species. In some instances, samples were omitted from sequencing when the library prep procedure failed to produce adequate cDNA. The TruSeq Stranded Total RNA Library Prep Kit Gold (Illumina, San Diego, CA, United States) was utilized to generate cDNA libraries with capture of coding and multiple forms of non-coding RNA. The prepared cDNA libraries were sequenced on the Illumina NextSeq 500 instrument using Illumina’s NextSeq 500/550 High Output v2.5 kit. Each sequencing run anticipated 33–50 million reads per sample, with paired-end sequencing of 76 base pairs (bp) in the forward and reverse direction. The median percentage of total reads uniquely mapped to the reference genome ranged from 66.9% to 78.2% across all species. All reads were mapped to the respective reference genome for each species using Omicsoft Software OSA4 (Omicsoft, Cary, NC, United States), which are referenced in Table 2.

**TABLE 2:** Transcript mapping information across species.

	Mouse	Rat	Dog	Monkey
Assembly	Mouse.B38	Rat.B6.0	Dog.CanFam3.1	Macaca_fascicularis_5.0
Protein-coding	21,968	22,250	19,856	20,815
Non-protein coding	7,913	10,633	4,724	6,244
Average alignment	76.369%	66.854%	78.170%	75.215%
Average read depth	97,033,160	119,424,748	162,362,994	86,051,784

### 2.5 *In vitro* assay for recombinant IL-22 activity

Frozen primary human (Cat # HMCPP5), cynomolgus monkey (Cat # MKCP10), rat (Cat # RTCP10), and mouse hepatocytes (Cat # MSCP10) were purchased from ThermoFisher Scientific. Individual vials of cells for each species were thawed and plated on 96-well collagen-coated plates (Corning^®^, 354407) according to the ThermoFisher protocol. Hepatocytes were plated at a density of 50,000 cells/well and were incubated under standard cell culture conditions of 37°C and 5% CO_2_. Recombinant interleukin-22 (rIL-22) specific for human (Cat # 782-IL-010), rat (Cat # 1316-RL-010), and mouse (Cat # 582-ML-010) were purchased from R&D Systems and used as a reference positive control for STAT3 activation. After one overnight incubation, human and cynomolgus monkey hepatocytes were treated for 30 min with human rIL-22 (rhIL-22) in 7 serial 8-fold dilutions ranging from 600 nM to .0023 nM. Rat and mouse hepatocytes were treated with rIL-22 of respective species, in 7 serial 8-fold dilutions ranging from 600 nM to .0023 nM, for 2 different time points of 15 and 30 min. Published literature suggested that rat hepatocytes may need a longer incubation time to have a STAT3 phosphorylation response from IL-22 signaling (Lee et al., 2018). Based on this, we conducted additional experiments using rat primary hepatocytes and incubated with rat rIL-22 for 30 min, 6 h, and 24 h.

At the end of incubation periods, media was removed from the wells and replaced with Tris lysis buffer, a component of the Phospho-STAT3(Tyr705) detection kit purchased from Meso Scale Discovery (MSD, Cat #K150SVD-2). Following the kit protocol, cells were lysed for 30 min at 4°C, and then lysates were added to the MSD plate for subsequent detection of phosphorylated STAT3 (pSTAT3).

### 2.6 Data analyses

FastQ files were generated and uploaded to Array Studio (OmicSoft, Cary, NC, United States) for reference genome alignment and generation of transcript count data for all samples (Table 2). Transcripts were assessed and normalized using DESeq2 (Love et al., 2014).

Within each species, tissue samples were averaged and principal component analysis (PCA) clustering was applied using R (R_Core_Team, 2017) and R Package PCATools (Blighe and Lun, 2022). Hierarchical clustering and data visualization was performed using R Package GGplot2 (Wickham et al., 2016). Gene enrichment analyses were performed using EnrichR (Chen et al., 2013). Data visualization for pSTAT3/IL-22 and flavin-containing monooxygenase (FMO) data was performed using GraphPad Prism version 9.0.0 for Windows (San Diego, California United States, www.graphpad.com). Transporter heatmaps were generated using normalized Z-scores calculated from FPKM values per gene using the R package pheatmap (Kolde, 2019).

## 3 Results

### 3.1 Clinical pathology and histology

In all four species, hematology (Supplementary Material S1) and clinical chemistry (Supplementary Material S2) parameters were evaluated and confirmed to be within the normal range. Similarly, there was no microscopic evidence of disease or unexpected background/spontaneous changes in any of the tissues evaluated. These results confirmed that animals used for this study were healthy and without any background disease or organ dysfunction.

### 3.2 Evaluating tissue mRNA expression differences

To evaluate the expression profile of genes at the transcript level in different tissues/organs in each species, RNA-sequencing data were analyzed to calculate fragments per kilobase of exon per million mapped fragments (FPKM) values for both protein and non-protein coding genes. The number of transcripts evaluated depended on the species and reference transcriptome assemblies used to map the sequenced reads (Table 2).

After calculating the median FPKM values across the biological replicates, transcripts were defined as “expressed” where FPKM ≥ 1. Across all the species evaluated, the number of transcripts expressed in a tissue ranged from 11,550 to 19,318 and not surprisingly was found to differ based on tissue and/or species. In all species, similarities for relative gene expression were observed for specific tissues. Consistent with other studies (Yu et al., 2014), the liver, heart, and skeletal muscles express relatively less genes than other tissues (Supplementary Figures S1A, B, S2A, B). Differences in the average expression across all expressed transcripts were also observed between tissues (Supplementary Figures S3A, B, S4A, B).

**FIGURE 2:**
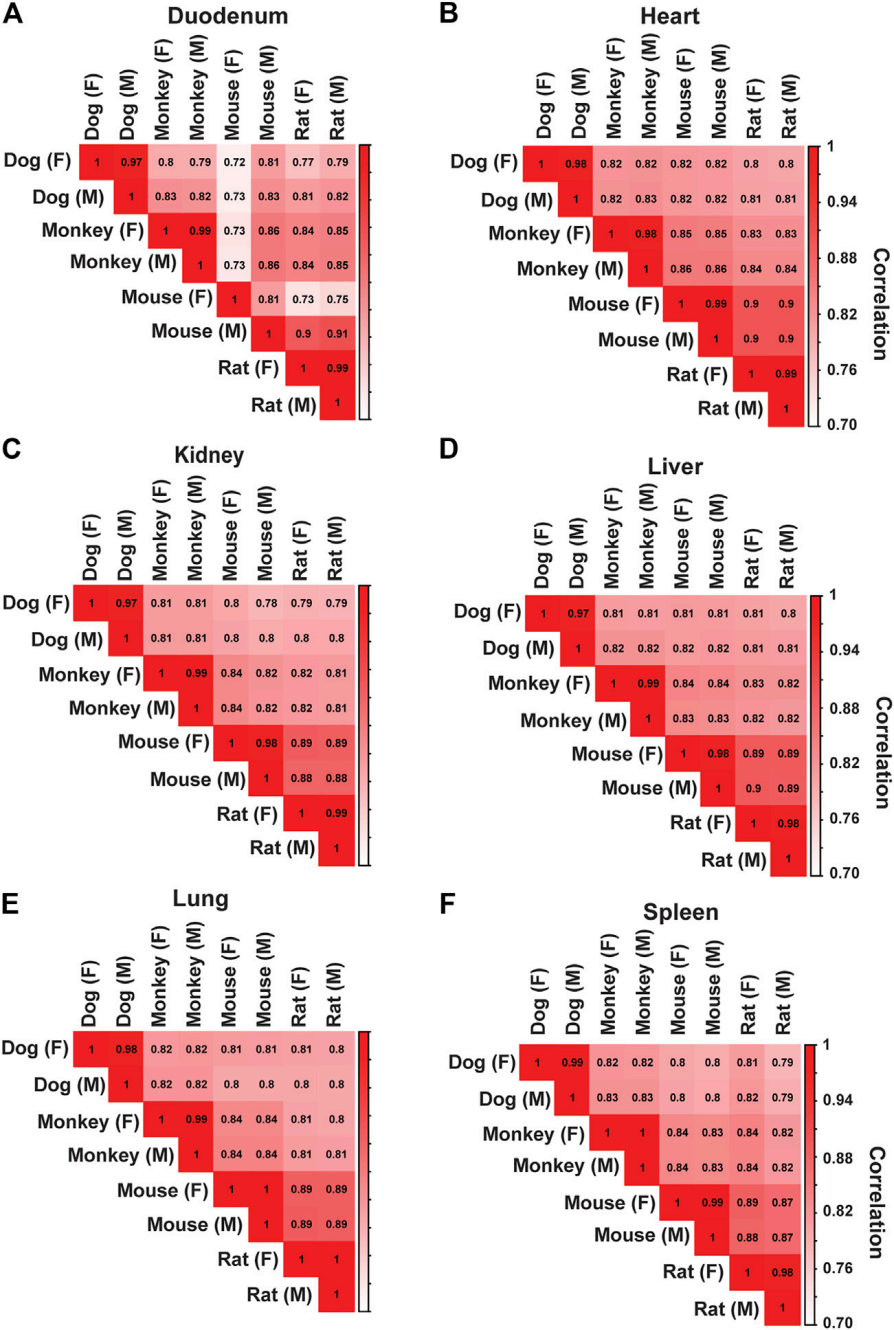
Correlation Analysis Across Species for Major Toxicity Organs. Correlation analysis was performed to assess species and sex differences across tissues. In both males (M) and females (F), spearman correlation coefficient values were generated across all species in **(A)** Duodenum, **(B)** Heart, **(C)** Kidney, **(D)** Liver, **(E)** Lung, and **(F)** Spleen.

Protein-coding genes for each species ranged from 19,856 (dogs) to 22,250 (rat), and non-protein coding genes ranged from 4,724 (dog) to 10,633 (rat). Average read depth ranged from 86,051,784 (monkey) to 162,362,994 (dog). To better interrogate species differences across tissues, we filtered and evaluated 13,072 protein-coding genes that were annotated across all species in this study. Frequent target organs of drug-induced toxicity were selected for spearman correlation analysis in both males and females: duodenum, heart, kidney, liver, spleen, and lung. Correlation analyses were performed across species (Figures 2A–F) and across all target organs (Supplementary Figure S5). Overall, we observed a strong relative correlation between similar tissues in both males and females. Other tissues were also assessed using spearman correlation (Supplementary Material S3).

### 3.3 Clustering analyses across species and tissues

**FIGURE 3:**
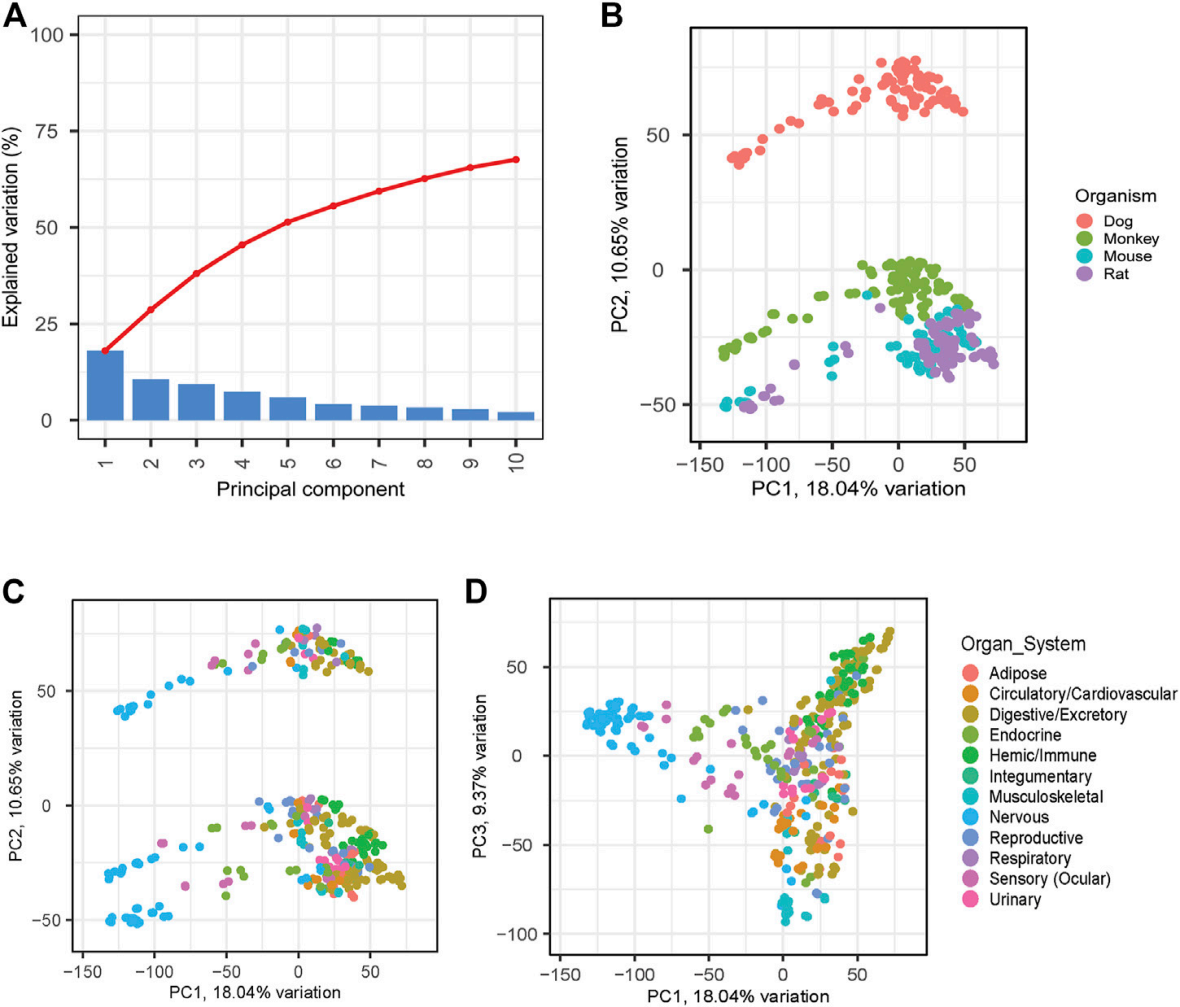
PCA Analyses to Identify Relationships between Species and Samples. PCA analyses was applied across all matching samples between species. **(A)** Scree plot shows explained variation across the first 10 PCs. PC1 versus PC2 was plotted highlighting species differences **(B)** and tissue differences **(C)**. PC3 was also evaluated to evaluate additional tissue differences **(D)**.

Principal Component Analysis (PCA) was used to evaluate associations across the samples. Principal components (PC) 1, 2, and 3 were observed to explain approximately 18, 11, and 9 percent of the variation within the data, respectively. (Figure 3A). PC2 shows distinct clustering across species and indicates a relatively closer relationship between the rodents (mouse and rat) and clear separation from dog and monkey (Figure 3B). Interestingly, dog was observed to have the strongest separation on PC2. We further interrogated PC1 by annotating tissues and nervous system-related tissues were responsible for much of the variation on PC1 (Figure 3C). PC3 was also evaluated resulting in a strong clustering of hemic and immune system tissues, which may be driven by common hematopoiesis processes (Short et al., 2019; Gomes et al., 2021) (Figure 3D).

PCA loading analyses were performed across the top 5 PCs to help identify genes responsible for the explained variance across the PCs (Supplementary Material S4). The top 500 gene responses for the negative PC1 loading were extracted and assessed using gene set enrichment analysis (GSEA). Pathways related to GABA receptor activity and Benzodiazepine were identified among the top hits and are known to be associated with central nervous system function (Griffin et al., 2013; Wu and Sun, 2015) (Table 3). In both mouse and rat, similar analyses were performed on a smaller subset of tissues and identified the brain samples as having the most distinct cluster on PC1 (Sollner et al., 2017). Due to the large number of tissues and species, we expect clustering analyses to be complex with distinct PCs being responsible for driving variation between distinct tissue groups and species.

**TABLE 3:** Gene enrichment analysis for negative PC1 loading.

Term	Overlap	*p*-value	Adjusted *p*-value	Odds ratio
Transmitter-gated ion channel activity	16/34	2.67E-17	1.00E-14	35.78
GABA receptor activity	13/22	5.17E-16	9.67E-14	57.81
GABA-gated chloride ion channel activity	10/13	2.33E-14	2.91E-12	132.63
Ligand-gated anion channel activity	11/18	5.81E-14	5.43E-12	62.64
Ligand-gated channel activity	13/30	1.04E-13	7.21E-12	30.59
GABA-A receptor activity	11/19	1.35E-13	7.21E-12	54.81
Glutamate receptor activity	11/19	1.35E-13	7.21E-12	54.81
Ligand-gated ion channel activity	13/31	1.75E-13	8.16E-12	28.89
Neurotransmitter receptor activity involved in regulation of postsynaptic membrane potential	11/21	6.02E-13	2.50E-11	43.84
Ionotropic glutamate receptor activity	11/17	1.45E-12	5.42E-11	56.83

### 3.4 Hierarchical clustering across tissues

**FIGURE 4:**
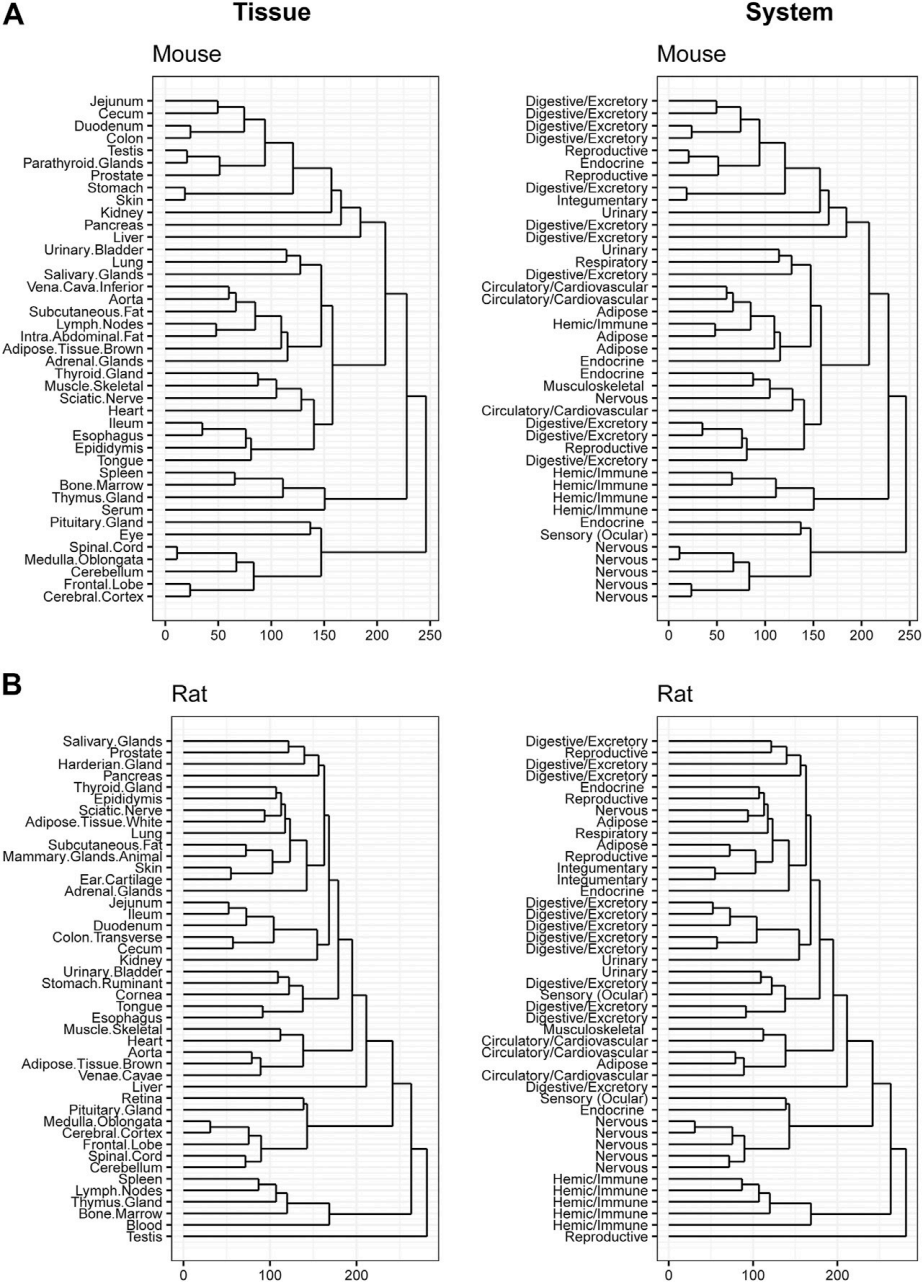
Hierarchical Clustering of Tissues in Mouse and Rat. Dendrogram to visualize hierarchical clustering of male tissues (left) in mouse **(A)** and rat **(B)**. These tissues were also mapped to broader organ/system classifications (right).

**FIGURE 5:**
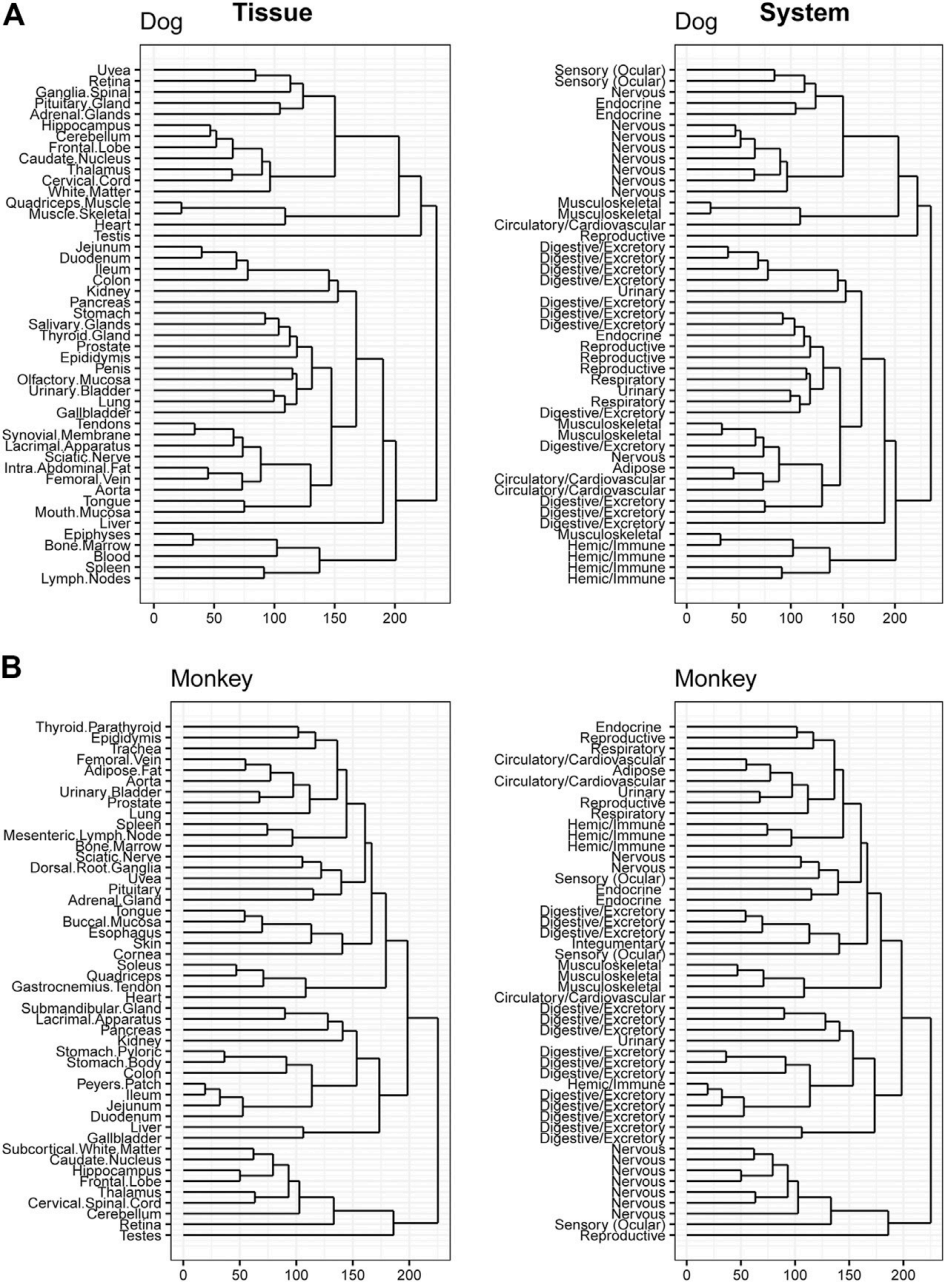
Hierarchical Clustering of Tissues in Dog and Monkey. Dendrogram to visualize hierarchical clustering of male tissues (left) in Dog **(A)** and Monkey **(B)**. These tissues were also mapped to broader organ/system classifications (right).

Hierarchical clustering was performed to confirm data quality and evaluate tissue differences within species. Tissue clustering was performed on the male tissues and annotated with the organ classifications for both rodents (Figures 4A,B), and large animals (Figures 5A,B). Similarly analyses were performed on female tissues in rodents (Supplementary Figures S6A,B) and large animals (Supplementary Figures S7A,B). Due to the large number of genes that are expressed uniquely in the testes (Djureinovic et al., 2014), a strong diversification between testes and other tissues in rat, dog, and monkey was observed. Related tissues from the same organ system, such as nervous, digestive, and immune tissues, generally clustered together.

### 3.5 Case examples to evaluate correlation of gene expression profile with known species and sex differences in pharmacological and/or metabolic responses

#### 3.5.1 IL-22 receptor signaling

**FIGURE 6:**
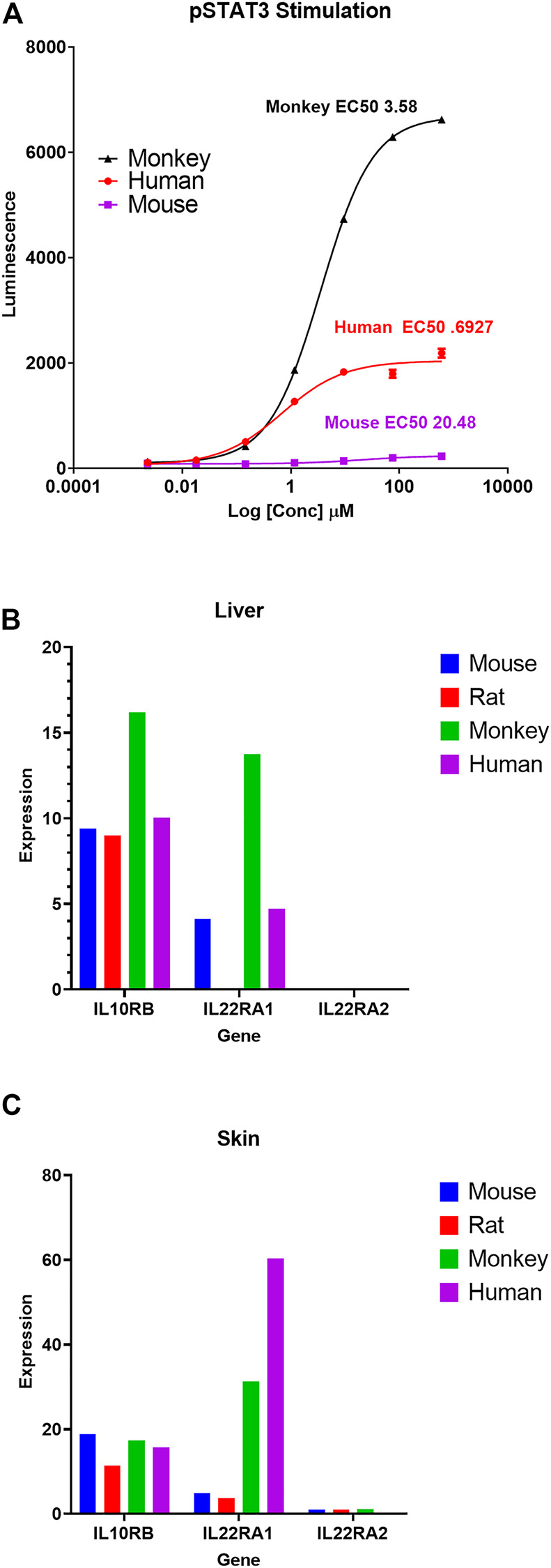
Differential pSTAT3 stimulation and IL-22 receptor genes expression across species. **(A)** pSTAT3 stimulation dose curves in response to recombinant IL-22 (rIL-22) in mouse, rat, monkey, and human primary hepatocytes. Species-specific rIL-22 was used for rat and mouse hepatocytes. rhIL-22 was used for monkey and human hepatocytes. For rat, there was no signal, so no value is shown in the graph **(A)**. Average FPKM expression of IL-22-receptor genes (IL10Rβ, IL-22RA1, and IL-22RA2) in Liver **(B)** and Skin **(C)** for mouse, rat, monkey, and human. Additional details on this case example are summarized in Table 4.

As a case example, the gene expression atlas was used to understand reported species difference in interleukin 22 (IL-22) signaling and associated changes observed in toxicity studies. IL-22 cytokine is a member of the IL-10 family and is recognized by IL-22R, a heterodimeric transmembrane receptor complex composed of IL-22R1 and IL-10Rβ (Xie et al., 2000). IL-22 activates STAT3 signaling cascades and mediates multiple cellular responses including induction of proliferative and anti-apoptotic pathways, as well as production of anti-microbial molecules (Sabat et al., 2014). An IL-22 recombinant fusion protein (UTTR1147A), that consists of the human cytokine IL-22 with the Fc portion of a human IgG4, was shown to have species differences of *in vitro* STAT3 activation potential (a biomarker of target engagement and histological changes in target cells) from preclinical toxicology studies (Lee et al., 2018). Specifically, rats were shown to be less sensitive than monkeys to IL-22 mediated pSTAT3 phosphorylation *in vitro* (hepatocytes) and for expected pharmacology related clinical pathology changes (increased serum fibrinogen and C-reactive protein) as well as skin toxicity (specifically epidermal hyperplasia) in repeat-dose toxicity studies. This difference in sensitivity was associated with an ∼10-fold difference in NOAEL levels (for skin toxicity) between rats and monkeys in 11-week repeat-dose toxicity studies (Table 4). Adverse skin reactions (dry skin, erythema, and pruritis) were also reported in healthy human volunteers and ulcerative colitis patients dosed with UTTR1147A in a Phase 1b study (Al-Bawardy et al., 2021). To further evaluate and confirm species differences in sensitivity for IL-22 signaling, we tested the *in vitro* pharmacologic activity of recombinant IL-22 (rIL-22) in primary hepatocytes isolated from mice, rats, dogs, monkeys, and humans. Species-specific rIL-22 was used for rat and mouse hepatocytes. rhIL-22 was used for monkey and human hepatocytes. Based on the EC50 values for STAT3 activation (Figure 6; Table 4), r-IL-22 was more potent in cynomolgus monkeys compared to mice and rats. In rat cells, IL-22 failed to produce a pSTAT3 signal even with extended duration of incubation (up to 24 h) compared to other species (30 min) tested.

**TABLE 4:** Summary of species sensitivity difference for IL22 signaling and associated pharmacology and safety findings.

	Repeat dose toxicity study (11 weeks) or phase I trial data h IL-22FC IG fusion protein (Lee et al 2018; Al-Bawardy et al 2021)			STAT3 activation signal in primary hepatocytes for rhIL22 and hIL-22 fusion protein^a^ (Lee et al 2018)	EC50 value for rIL22 induced pSTAT3 in hepatocytes
	Clinical pathology^b^	Skin toxicity	NOAEL		
			Dose (AUC)		
Monkey	CRP ↑	Epidermal hyperplasia at ≥75 µg/kg (IV, once every 2 weeks)	15 μg/kg (333 ng.day/ml)	+	3.5 nM
	Fibrinogen ↑				
Rat	No change	Epidermal hyperplasia at 500 µ/kg (IV, twice weekly)	150 μg/kg (2,370 ng.day/ml)	-	No signal
Mouse	--	--	--	+	32 nM
Human	--	Dry skin, erythema, and pruritis (30–90 μg/kg either biweekly or monthly)	--	+	.69 nM

^a^ At 30-min timepoint (Western blot data for pSTAT).

^b^ Changes related to target engagement.

+, Elevations in STAT3 phosphorylation; -, No STAT3 phosphorylation; --, no data.

Based on this background information, we used our comprehensive body atlas data to evaluate if the lower potency in rodents compared to monkeys is correlated with differential basal expression of IL-22R genes (mainly IL-22RA1 and IL-10Rβ) in two representative key IL-22 target tissues (liver and skin). IL-22RA1 expression was highest in monkeys in both liver and skin, compared to rodents. Interestingly, there is little to no expression of IL-22RA1 in rat, specifically in the liver (Figure 6). IL-10Rb expression levels were consistent across mouse and rat liver but significantly increased in monkeys. Based on the GTEx database, the expression of IL-22RA1 in human skin appears to be relatively higher than monkeys.

#### 3.5.2 Flavin-containing monooxygenases (FMO) expression levels

**FIGURE 7:**
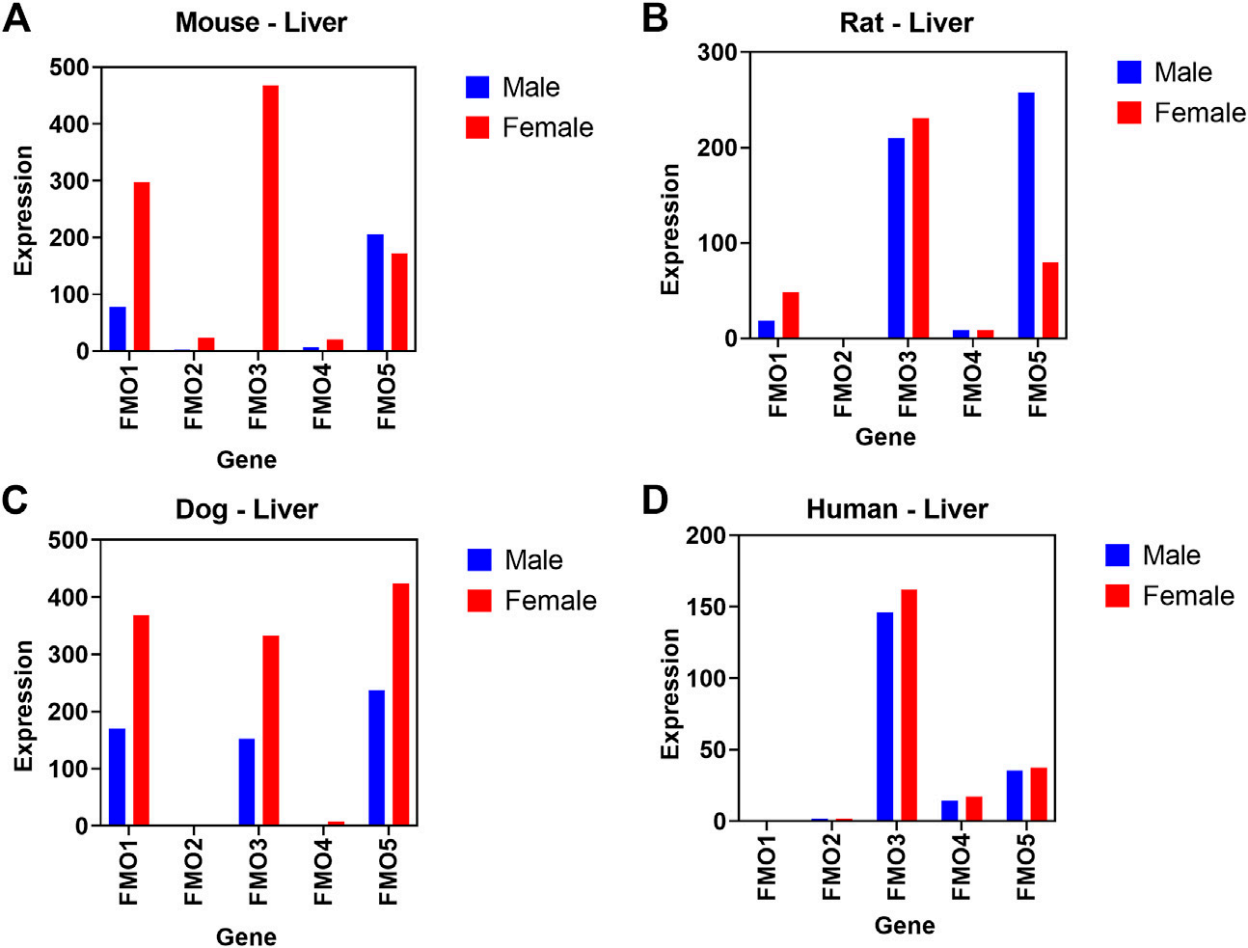
Relative difference in transcript level expression of Flavin-containing monooxygenases (FMO) genes. Sex differences between male (blue) and female (red) are shown across 5 different FMO isoforms in mouse **(A)**, rat **(B)**, dog **(C)**, and **(D)** human liver.

In another case study, we evaluated if basal transcript expression in our database correlates with known sex-related differences in expression and activity of Flavin-containing monooxygenases (FMO). In both mice and dogs, much higher levels of FMO1 and FMO3 expression were detectable at transcript levels in females compared to males (Figure 7). A comparison of transcript level expression of FMO isoforms in preclinical species in our comprehensive body atlas data supported previous reports of species and sex dependent relative FMO1 and FMO3 expression levels (Falls et al., 1995; Ripp et al., 1999). Interestingly, we did not observe these sex-related differences in humans.

#### 3.5.3 DDI transporter evaluation in liver, kidney, and heart

**FIGURE 8:**
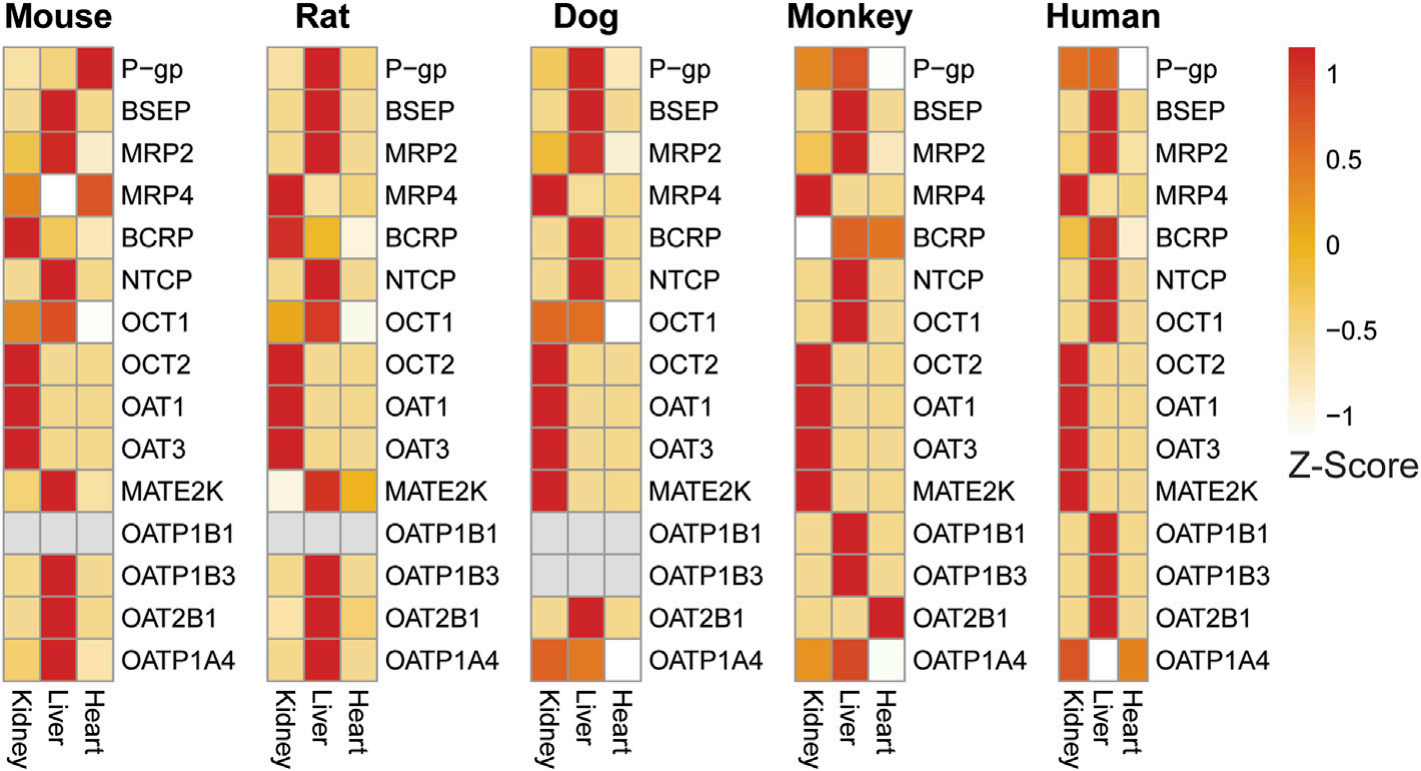
Expression heat map of key transporters in the liver, kidney, and heart of male preclinical species and humans confirms tissue-specific expression of liver and kidney transporters across species. Heart was included as a general negative control as few transporters are reported to be highly expressed there. Expression patterns are generally consistent across species with some notable exceptions.

To further demonstrate utility of our RNAseq database, we compared relative expression levels of key drug efflux transporters (P-gp, BCRP, MRP2, MRP4, MATE2K) and drug uptake transporters (OATP1B1, OATP1B3, OAT2B1, OATP1A4, OAT1, OAT3, OCT1, OCT2, BSEP, and NTCP) across liver, kidney and heart (negative control tissue) in males (Figure 8) and females (Supplementary Figure S8). The 2018 International Transporter consortium (ITC) recommended these transporters as being of key importance in drug development (Zamek-Gliszczynski et al., 2018). In addition, we compared the relative expression of these transporters in human tissues using the GTEx human expression body atlas with preclinical species.

Drug transporters known to be specific or enriched in liver such as BSEP, MRP2, OCT1, OATP1B1, OAT1B3, OAT2B1, OATP1A4 showed higher expression in liver samples compared to kidney and heart (Figure 8) confirming the expected tissue-specific abundance across species. Higher expression of both OATP1B1 and OATP1B3 is unique to both humans and monkey liver (Wang et al., 2015). In addition, strong expression of OATP1A4 (rodent ortholog of human OATP1B1) was detected in both mouse and rat liver. Similarly, our database confirmed higher levels of transporters specific or enriched in kidney such as OAT1, OAT3, and OCT2, MRP4 compared to liver and heart in all four preclinical species and were also comparable to respective human tissue. In heart, which was included as a negative control tissue for transporters, expression of the majority of transporters evaluated were either very low or not detected (except for P-gp and MATE2K in mouse and BCRP and OAT2B1 in monkey).

Further comparison of the expression of a few selected transporters between species, sex, and tissues demonstrated an overall good correlation with published information. For example, species and tissue specific distribution of organic cation transporters (OCTs) are well documented (Bleasby et al., 2006). OCT1 enrichment was limited to the liver in human and monkeys, whereas in rat, mouse and dog expression is detected in both liver and kidney (Figure 8). Other than OCT1, the expression of other renal transporters evaluated appeared to be comparable across preclinical species and human at the transcript level. BCRP expression was more highly expressed in male rats uniquely, but this difference was not found in other species (Figure 8; Supplementary Figure S8).

Interestingly, the expression profile of the multidrug and toxin extrusion protein (MATE2K) was different in rodents from other species. In dog, monkey, and human, high expression of MATE2K was observed in kidneys, whereas in rats and mice, high expression is detected only in liver, but not in kidney.

## 4 Discussion

Many applications of this comprehensive preclinical species transcriptomic database can be envisioned to improve or expedite drug discovery and development. These data could be used to predict potential target tissues of toxicity in preclinical species as part of a target safety assessment and to design better early toxicology studies (Roberts, 2018). The same exercise can reasonably be conducted for confirmed or predicted off-target drug interactions (Van Vleet et al., 2019). In addition, these data could serve to guide therapeutic target selection. Likewise, species-specific expressions may be helpful in understanding species-specific sensitivity to certain drugs and the human relevance of preclinical findings (Spurgeon et al., 2020). The translation of preclinical toxicity findings to humans, when mechanism is understood, could also be better predicted with these body atlas expression data. Finally, body atlas gene expression may serve, in part, for selecting or justifying the most appropriate preclinical toxicity species (Prior et al., 2020).

Several RNAseq body atlas databases from preclinical species have been previously developed. Some of these contain data from species which are of importance for drug safety evaluation such as mouse, rat, and monkey (Perry et al., 2012; Li et al., 2017; Ji et al., 2020; Thiemeyer et al., 2021). However, significant strain-related differences in drug-related response and sensitivity have been noted as well, reflecting the need for RNAseq databases in the most common species and strain used for preclinical toxicology assessment of drugs (Annas et al., 2013; Hoffmann et al., 2017). In this body of work, we created an RNAseq body atlas in the strains of mouse (CD1), rat (Sprague Dawley), dog (Beagle), and monkey (Cynomolgus) most used in preclinical drug safety studies.

Prior to this work, no comprehensive CD1 mouse body atlas RNAseq databases had been established. CD1 mice are the most common mouse strain for drug toxicology studies (Annas et al., 2013). Our sequencing efforts identified 21,968 protein coding and 7,913 non-coding genes in CD1 mice (Table 2). More limited CD1 mouse tissue profiling efforts have been reported previously. For example, Kahr et al. (2011) evaluated transcriptional expression differences between right and left atria in 3 strains of mice, including CD1, compared to humans. C57BL/6 mice have been used to create several RNAseq expression databases. For example, Hounkpe et al. (2021) published a RNAseq database from 52 to 14 different normal tissues in humans and C57L/6 mice, respectively. The database was used to identify 2176 and 3277 housekeeping genes in humans and mice, respectively. Likewise, Li et al. (2017) created a comprehensive, 17 tissue, RNAseq database from C57BL/6 mice to identify 4,781 housekeeping genes. In this work, 27 consistently and highly expressed genes were identified as reference controls and 22,196 protein coding and 21,432 total non-protein coding genes were identified. These values are similar to those we report above for CD1 mice. Finally, a 2017 study created a C57BL/6 mouse and Han Wistar rat RNAseq Body Atlas from 13 tissues in each species (Sollner et al., 2017). This study showed that tissue samples from both species clustered similarly in the PCA space, had many genes with high sequence similarity with human orthologues, and had highly correlated tissue distribution profiles to humans.

Other rat RNAseq databases have been reported in Fischer 344 rats. For example, Yu et al. (2014) created a body atlas map across 11 organs at 4 different developmental stages and identified 40,064 genes and 65,167 transcripts with 31,909 alternatively spliced variants. Similarly, Ji et al. (2020) presented an RNAseq database in Fischer 344 rats that included 11 organs. The study annotated 15,852 transcripts from 11,715 genes. Here for the first time, we introduce an RNAseq body atlas from Sprague Dawley rats, one of the most common rat strains used in drug toxicology studies (Weber et al., 2011; Annas et al., 2013). Similar to the values reported by Yu and Ji (2014 and 2020) from F344 rats, we identified 22,250 protein coding and 10,633 non-protein coding genes (Table 2).

No full body tissue RNAseq databases currently exist in the public domain for dogs. In this work, we present the first dog RNAseq body atlas. Our effort found 19,856 protein coding and 4,724 non-protein coding genes in beagle dogs (Table 2). This work was conducted in the dog strain (Beagle) used for drug safety toxicology studies (Weber et al., 2011; Uchida et al., 2015). At the time of this publication, the only dog related RNAseq databases that exist are focused on tumor tissue. Thiemeyer et al. (2021) presented an RNAseq framework for characterizing canine prostate cancers based on 10 malignant (spontaneously occurring) and 14 non-malignant prostate tissues from mixed breed dogs. The study identified 4,098 differentially expressed genes in tumors and 49 altered signaling pathways in prostate tumors. Likewise, Kim et al. (2019) presented an RNAseq whole transcriptome database of canine mammary gland tumors, sequencing 197 tumors and matched controls. Microarray technology has also been used previously to profile normal dog (both beagles and mixed breed dogs) tissue gene expression in 10 tissues including: liver, kidney, heart, lung, cerebrum, lymph node, spleen, jejunum, pancreas, and skeletal muscle (Briggs et al., 2011).

Multiple non-human primate body atlas databases have been reported, with atleast 2 containing transcriptome data from cynomolgus macaques, the most commonly used non-human primate (NHP) species for drug safety toxicology studies (Annick et al., 2015). In a comparative study of endangered primate species, Perry et al. (2012) reported a comprehensive database of RNAseq data from 21 tissues in 12 NHP and 4 other mammalian species. Cynomolgus monkeys were included in the database where an average of 5,721 gene sequences per species were identified. No relationship could be found between genetic diversity and endangered status. The Perry et al. database also contains liver transcriptomics for both the Indonesian and Mauritius strains of Cynomolgus Macaques, which are each used in toxicity studies and have been shown to have genetic differences related to differences in response to drugs/toxicants (Hoffmann et al., 2017). The database we introduce for Chinese cynomolgus macaques in this effort is the most comprehensive database to date with a total of 53 tissues represented. We identified 20,815 protein coding and 6,244 non-protein coding genes in cynomolgus macaques (Table 2). This value is similar to/greater than the average of 5721 primate gene sequences identified previously by Perry et al. (2012).

To illustrate the utility of our transcriptomic database, we evaluated if basal gene expression differences in preclinical tox species correlate with known species and/or sex differences in pharmacological response to IL-22 signaling and drug metabolizing enzyme (FMO) activity. In the IL-22 signaling case example, basal expression levels of both IL-22RA1 and IL-10Rβ genes in rat and monkey liver and skin samples (Figure 7) correlated well with known differential species difference in pharmacological and toxicological response for IL-22 signaling in *in vitro* pSTAT assay and *in vivo* repeat dose toxicology studies (summarized in Table 4). Interestingly, this difference was unique to IL-22R receptor complex and not observed for IL-22RA2, a naturally occurring IL-22 antagonist as it negatively regulates IL-22 signaling (Xu et al., 2001). IL-22RA2 transcript expression levels in liver and skin are relatively low, but comparable between mouse, rat and monkey. Further comparison to a publicly available human gene expression data base revealed that the IL22R expression level appeared to be comparable to monkeys, with relatively higher expression of IL22RA1 in both liver and skin. Overall, these results support a good correlation between reported species difference in the IL-22R mediated pharmacological response and basal expression of IL-22R genes at the transcript level compiled in our body atlas.

Species and sex differences in xenobiotic and drug metabolizing enzyme expression levels and activity are well known in human and other preclinical tox species (Martignoni et al., 2006; Waxman and Holloway, 2009). FMO enzymes catalyze the NADPH-dependent oxidation of a wide variety of nucleophilic compounds containing nitrogen, sulfur, and phosphorus moieties (Burnett et al., 1994; Krueger and Williams, 2005). A total of five different FMO isoforms have been identified (FMO1, 2, 3, 4, and 5), each with unique species- and tissue-dependent expression levels (Krueger and Williams, 2005). Among these five enzymes, FMO3 is the predominant and most studied isoform in adult human liver. In mice, FMO activity in liver microsomes is much higher in females than in males (Falls et al., 1995; Ripp et al., 1999). Specifically, FMO1 levels in female liver are shown to be 2 to 3 times higher compared to male mice and FMO3 expression are comparable to FMO1 in females, but undetected in males (Falls et al., 1995). However, FMO5 expression levels are comparable in both sexes. Mechanistically this sex-related difference in mice is attributed to male sex hormone- (testosterone) induced repression of FMO expression (Duffel et al., 1981). This sex-difference in FMO expression is not a mouse-specific phenomenon, because higher levels of FMO3 isoform are also observed in liver microsomes derived from mouse and dog compared to other laboratory species such as rats and rabbits (Ripp et al., 1999).

Using our body atlas data, comparison of transcript level basal expression of FMO isoforms in liver samples confirmed/supported these reports of species and sex related difference in FMO1 and FMO3 expression levels. As shown in Figure 8, in both mice and dogs, much higher levels of FMO1 and FMO3 expression were detectable in females compared to males. In humans (based on GTEX data), as expected FMO3 is the predominantly expressed isoform and expression level was comparable in both male and female liver samples. FMOs are known to catalyze oxidation of several drugs and chemicals into metabolites with either reduced or increased toxicological properties compared to parent compounds (Ripp et al., 1997; Krueger and Williams, 2005). Hence understanding of species, sex, and tissue level differences in the relative expression of FMO and potentially other drug metabolizing enzymes in preclinical toxicology species using our comprehensive body atlas data would provide valuable information in selecting relevant species for metabolism and toxicology studies. The two case examples discussed above, clearly support that the gene expression data we compiled is a useful resource to predict cross species biological differences and to improve preclinical pharmacology and toxicology experimental design and data interpretation.

The role of drug transporters in the disposition of drugs as well as their influence on pharmacokinetic (PK) based drug-drug interactions (DDI) and safety are well known (Chu et al., 2013; Zou et al., 2018). However, due to known species differences in tissue distribution, relative levels of expression in key tissues, and substrate specificity of these transporters, the extrapolation of data generated in preclinical species to humans can be challenging in some cases (Chu et al., 2013). Our RNAseq based tissue- and sex-specific transcriptomic database covering four preclinical tox species is expected to serve as a valuable tool to generate cross species quantitative mRNA level expression of drug transporters to understand species differences and improve allometry based prediction of PK and DDIs in humans. For example, OCT1 is shown to be expressed in liver in all preclinical tox species (mouse, rat, monkey) and human. However, OCT1 kidney expression was detected only in rodents, but not in the monkey and human (Bleasby et al., 2006). Correlating with this published report, our RNAseq data also show that OCT1 enrichment is limited to the liver in human and monkeys, whereas in rat, mouse and dog, expression is detected in both liver and kidney.

In addition to species differences, sex-related difference in expression of drug transporters are also reported. For example, relatively higher levels of breast cancer resistance protein (BCRP) expression were detected in the kidney of male rats compared to females (Tanaka et al., 2005). In our database, sex-related BCRP expression differences were observed in rat kidney (higher in males), but not other species.

A relative expression comparison of key drug uptake and efflux transporters in liver and kidney across species provided some good correlation with expected tissue specificity in liver and kidney as well as with previously reported species/tissue (OCT1) and sex (BCRP) specific abundance for the selected transporters. Even though mRNA expression may not always correlate with protein expression, potential inconsistencies exist when comparing expression levels in whole tissues versus key cells of interest within these tissues by either transcriptomics or proteomics approaches (Wang et al., 2015). We believe that a comprehensive RNAseq database will provide useful information to predict potential species differences in drug disposition and help select relevant preclinical species in early stages of transporter targeted drug development.

Data consistency is a significant consideration when developing a reference dataset. In this study, measures were taken wherever practical to procure samples in a way that optimized consistency. Certain decisions made to enhance workflow efficiency or to promote the 3R’s principles may have introduced a degree of variability to this dataset. Cynomolgus macaques utilized for tissue collections were protein naïve but had been dosed with non-protein test article in previous studies preceding a lengthy washout period. Similarly, male beagles utilized in our dataset had been included as vehicle-treated (DMA:PG:PEG-400 (20:40:40) animals in a terminal study immediately prior to tissue collection. Additionally, multiple methods were employed for tissue RNA extraction to optimize the quality and quantity of RNA isolated. For instance, these differences may be partially responsible for dog tissues showing relatively strong differentiation from other species based on PC2 analysis (Figure 3B). While this potentially introduced additional variability, it enabled inclusion of a wider array of species and tissue types within the dataset.

A separate effort to develop an RNAseq atlas of rodent gene expression noted relatively low gene expression variability when comparing the same tissue across separate individuals (Sollner et al., 2017). Based on this information, the decision was made to pool rodent samples to enhance workflow efficiency and reduce costs. Pooling biological replicates is a practice that has been evaluated previously in the context of RNA microarray gene expression analysis. While this approach may have implications on the statistical power, previous investigations have concluded that results from pooled samples have been adequate for detecting key gene changes at the group level (Peng et al., 2003). Likewise, pooling of samples can be a reasonable approach to RNAseq for detection of genes with low and moderate levels of expression to reduce costs and maintain power (Takele Assefa et al., 2020).

Developing a reference database of preclinical tissue gene expression offers a worthwhile opportunity to understand innate differences in target gene expression that have had limited characterization in preclinical species and enable users to further perform translational analyses with existing human expression databases. By performing RNAseq on a wide and diverse panel of tissues, we decrease the likelihood that there will be a future need to conduct additional tissue gene expression analyses, thereby reducing the overall animal requirement consistent with the 3Rs (replacement, reduction, and refinement) principle. This dataset provides valuable information to aid in species selection and experimental design, as well as in interpretation of study data, reducing costly study delays or repeat studies.

In summary, this work provides a comprehensive multi-tissue cross species database for male and female mice, rats, dogs, and monkeys. The species and strains used are of particular interest for drug safety assessment and prediction. The availability of these data will assist drug discovery and development and reduce the need to conduct of additional basal gene expression studies, reducing animal use. It is hoped that improved understanding of species-specific toxicities and preclinical to clinical toxicity translation will be significantly advanced for those applying this work.

## Data Availability

The original contributions presented in the study are publicly available. This data can be found here: https://www.ncbi.nlm.nih.gov/geo/query/acc.cgi?acc=GSE219045.

## Supplementary material

SUPPLEMENTARY FIGURE S1 Number of Transcripts Expressed Across Tissues. FPKMs were generated for transcripts across all samples. The number of expressed transcripts (FPKM ≥ 1) for each tissue was calculated for mouse **(A)** and rat **(B)**.

SUPPLEMENTARY FIGURE S2 Number of Transcripts Expressed Across Tissues. FPKMs were generated for transcripts across all samples. The number of expressed transcripts (FPKM ≥ 1) for each tissue was calculated for dog **(A)** and monkey **(B)**.

SUPPLEMENTARY FIGURE S3 Average Expression Across Tissues. The average Log_2_(FPKM) across all expressed transcripts in each tissue was calculated for mouse **(A)** and rat **(B)**.

SUPPLEMENTARY FIGURE S4 Average Expression Across Tissues. The average Log_2_(FPKM) across all expressed transcripts in each tissue was calculated across dog **(A)** and monkey **(B)**.

SUPPLEMENTARY FIGURE S5 Correlation Analysis Across Major Toxicity Organs. Spearman correlation analysis was performed to assess differences across duodenum, heart, kidney, liver, lung, and spleen in both males **(A)** and females **(B)**.

SUPPLEMENTARY FIGURE S6 Hierarchical Clustering of Tissues in Mouse and Rat. Dendrogram to visualize hierarchical clustering of female tissues (left) in mouse **(A)** and rat **(B)**. These tissues were also mapped to broader organ/system classifications (right).

SUPPLEMENTARY FIGURE S7 Hierarchical Clustering of Tissues in Dog and Monkey. Dendrogram to visualize hierarchical clustering of female tissues (left) in dog **(A)** and monkey **(B)**. These tissues were also mapped to broader organ/system classifications (right).

SUPPLEMENTARY FIGURE S8 Expression heat map of key transporters in the liver, kidney, and heart of female preclinical species and humans confirms tissue specific expression of liver and kidney transporters across species. Heart was included as a general negative control as few transporters are reported to be to be highly expressed there. Expression patterns are generally consistent across species and across sexes with some notable exceptions.
