# Supplementary figures and images for: Preclinical species gene expression database: Development and meta-analysis
Source: Front Genet. 2023 Jan 17;13:1078050. doi: 10.3389/fgene.2022.1078050 (PMC9887474; doi:10.3389/fgene.2022.1078050)

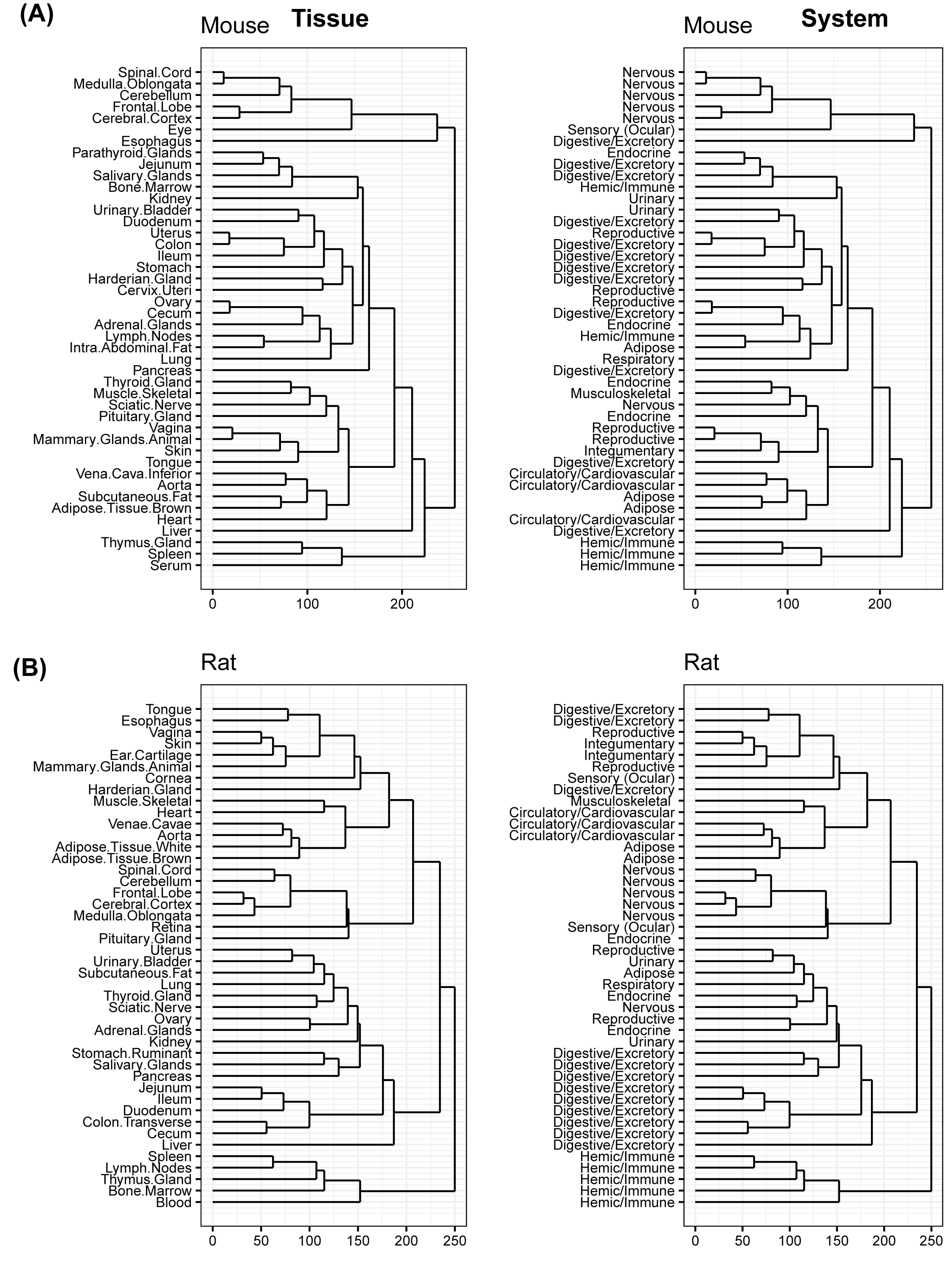

Supplement: Supplementary file 3 [file Image6.TIF]

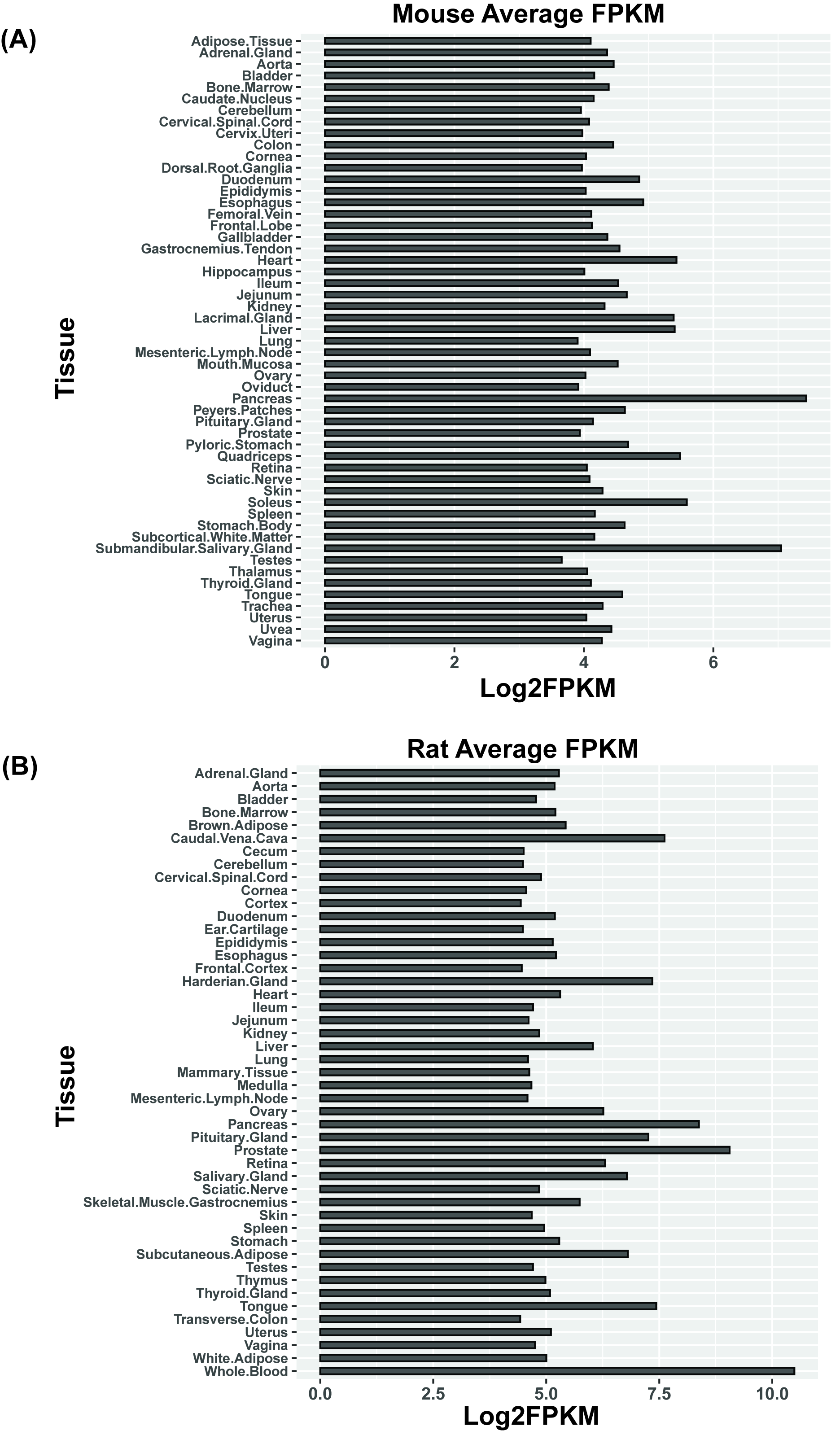

Supplement: Supplementary file 5 [file Image3.TIF]

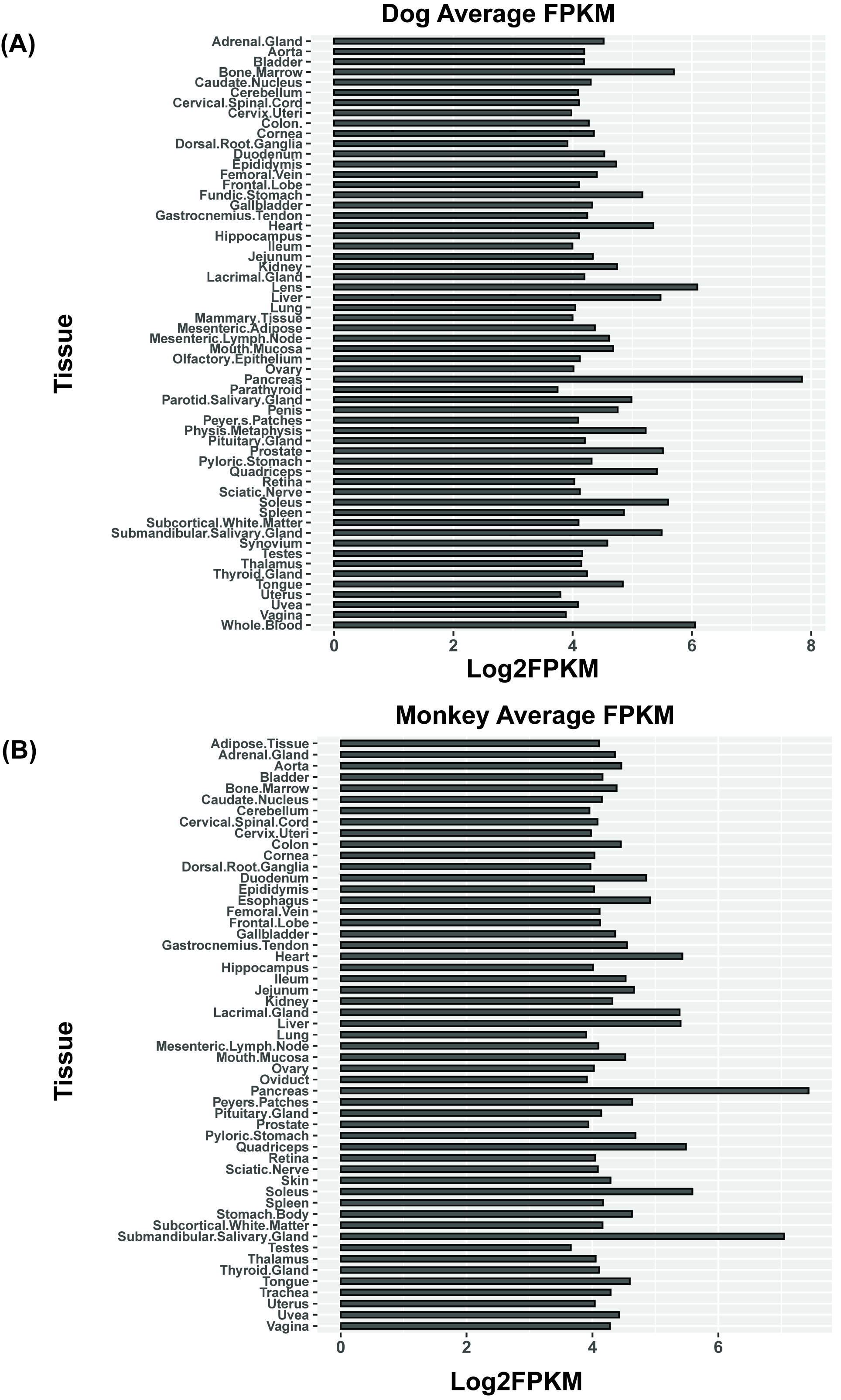

Supplement: Supplementary file 6 [file Image4.TIF]

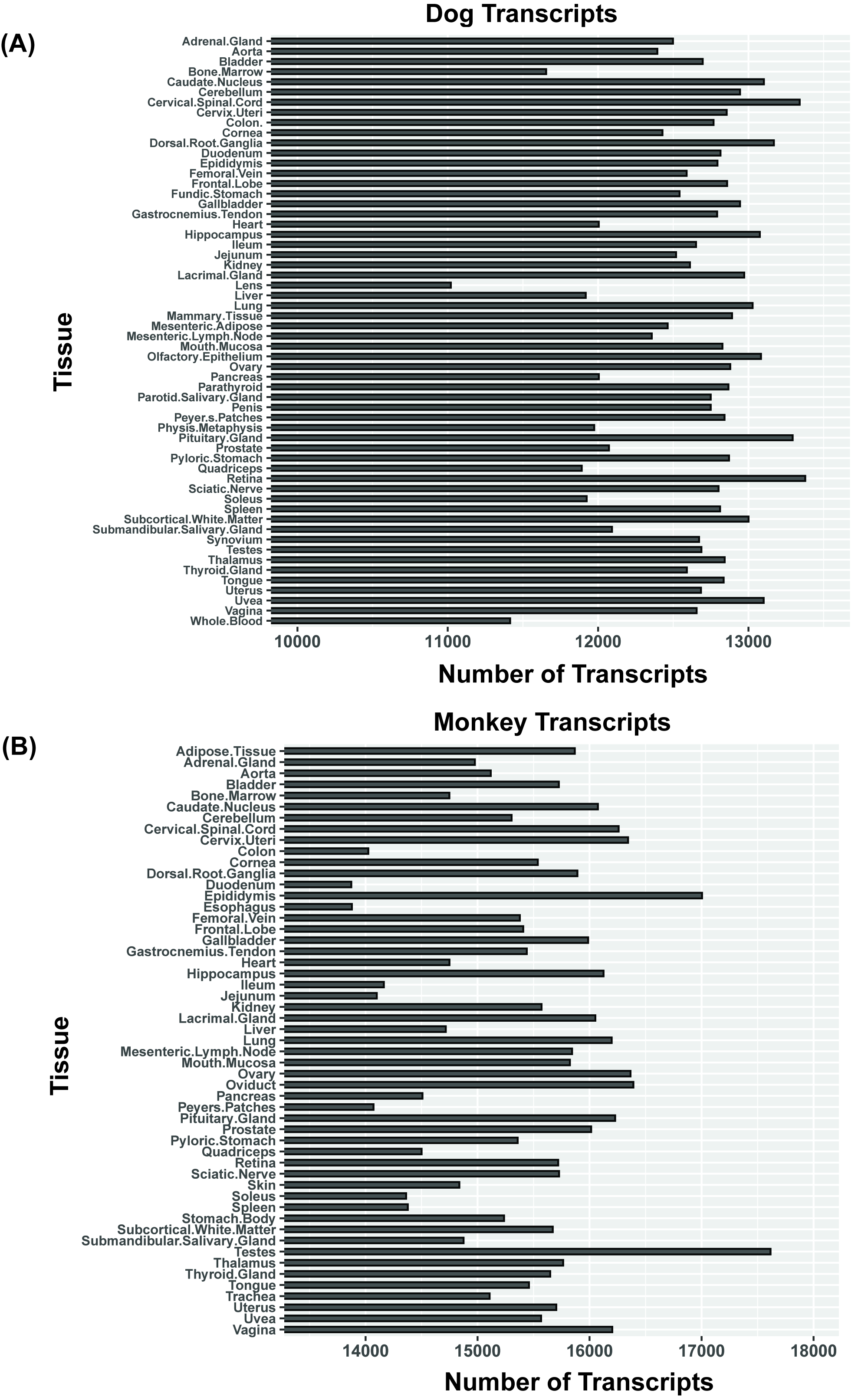

Supplement: Supplementary file 7 [file Image2.TIF]

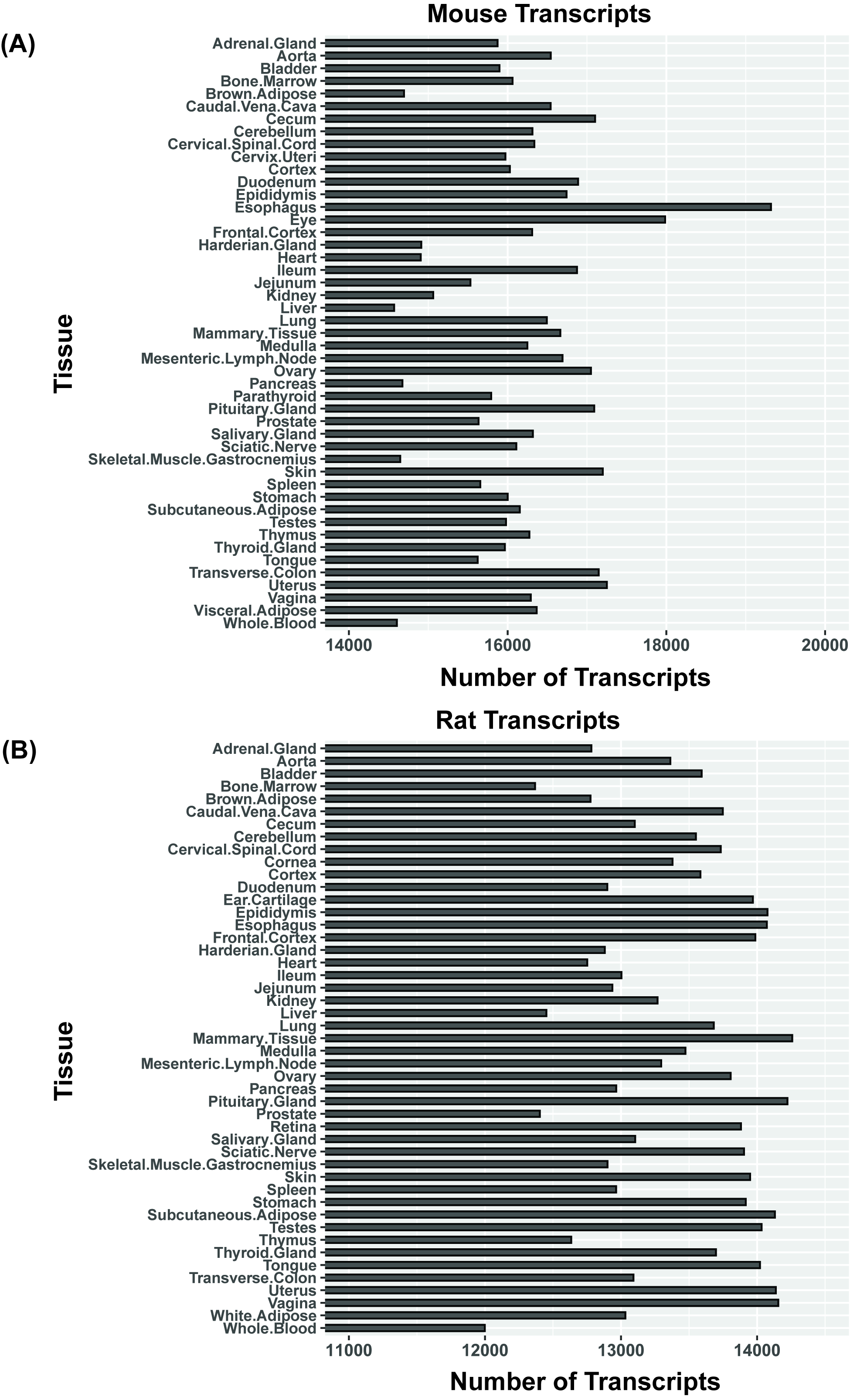

Supplement: Supplementary file 8 [file Image1.TIF]

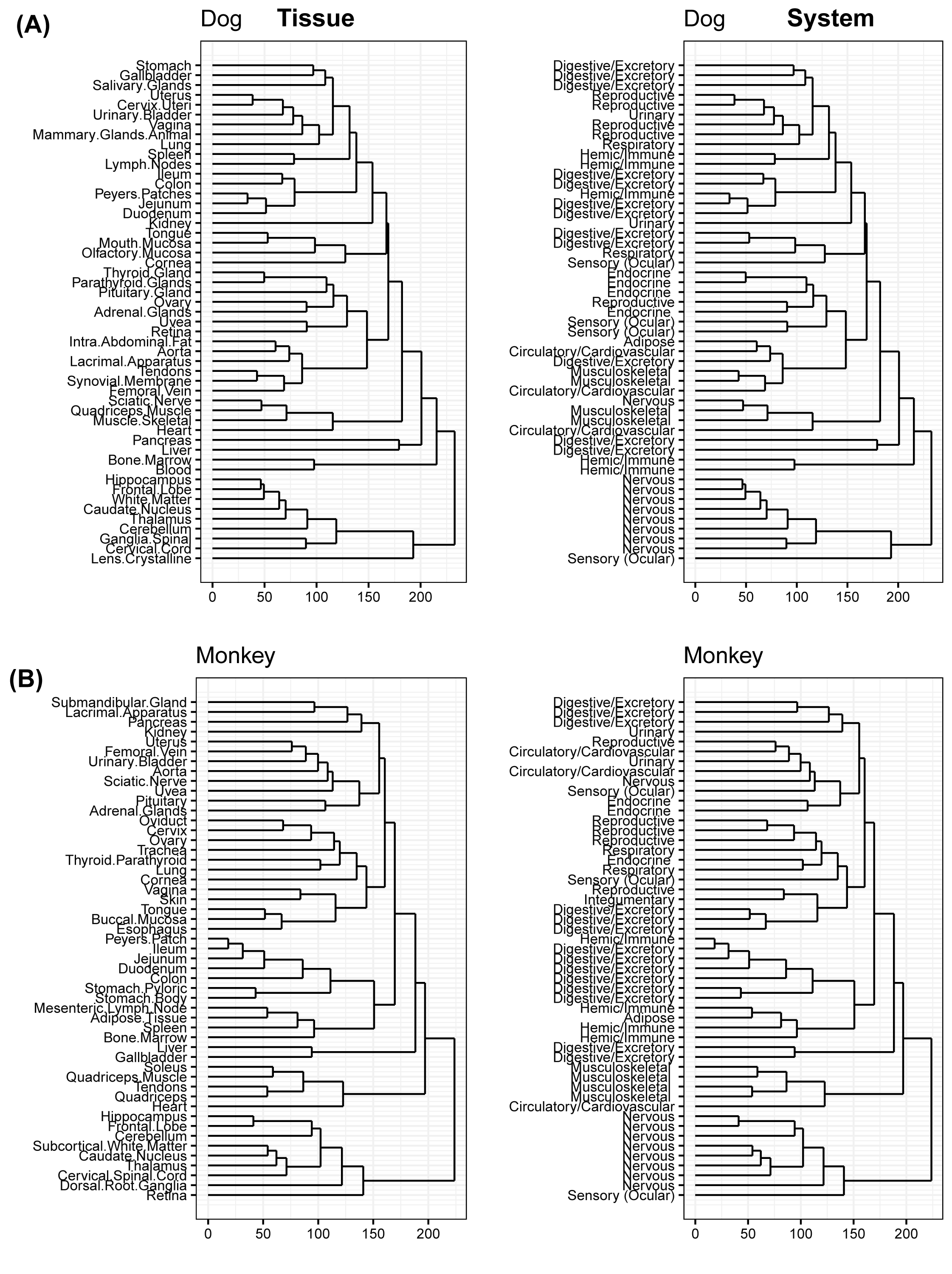

Supplement: Supplementary file 9 [file Image7.TIF]

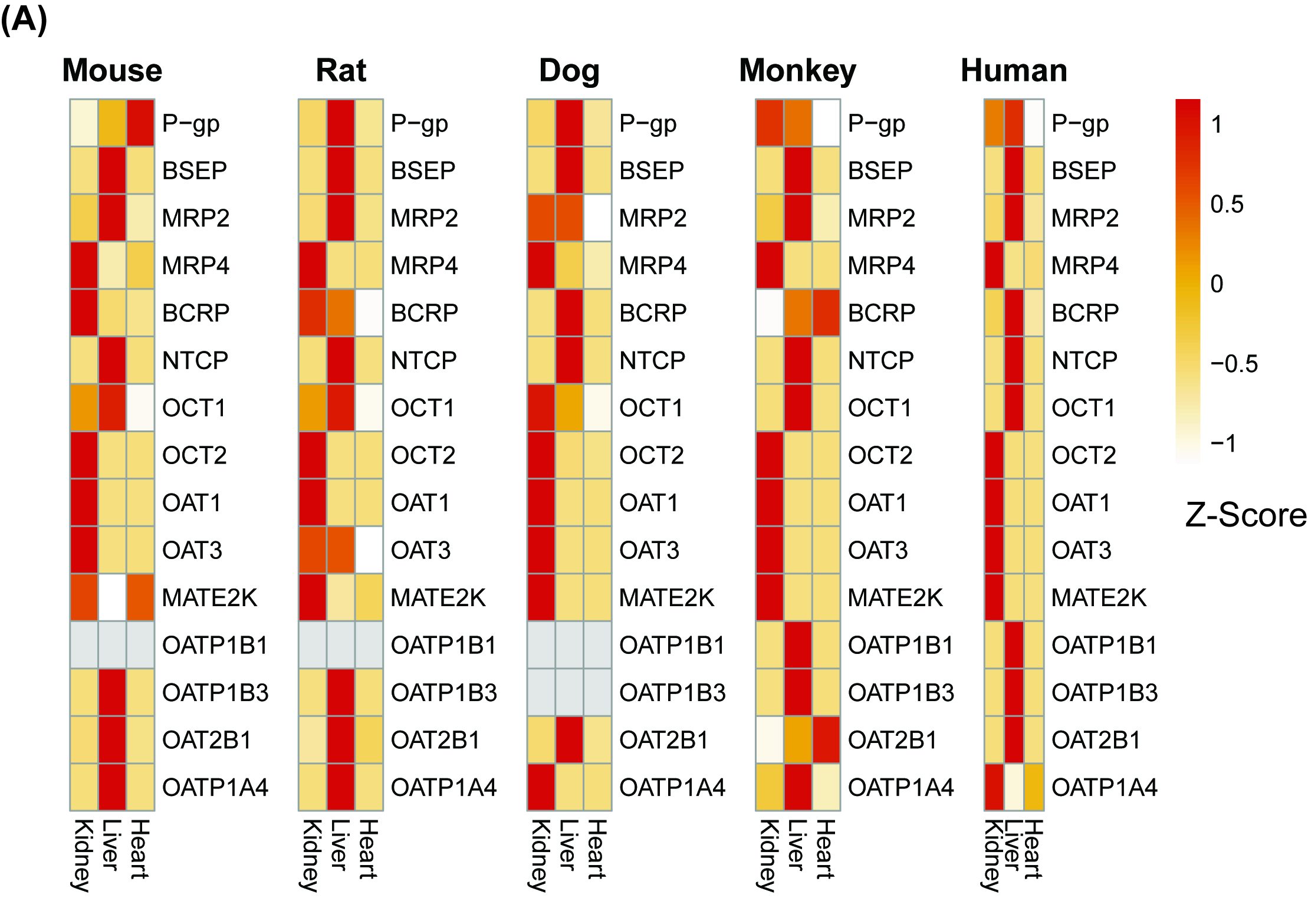

Supplement: Supplementary file 10 [file Image8.TIF]

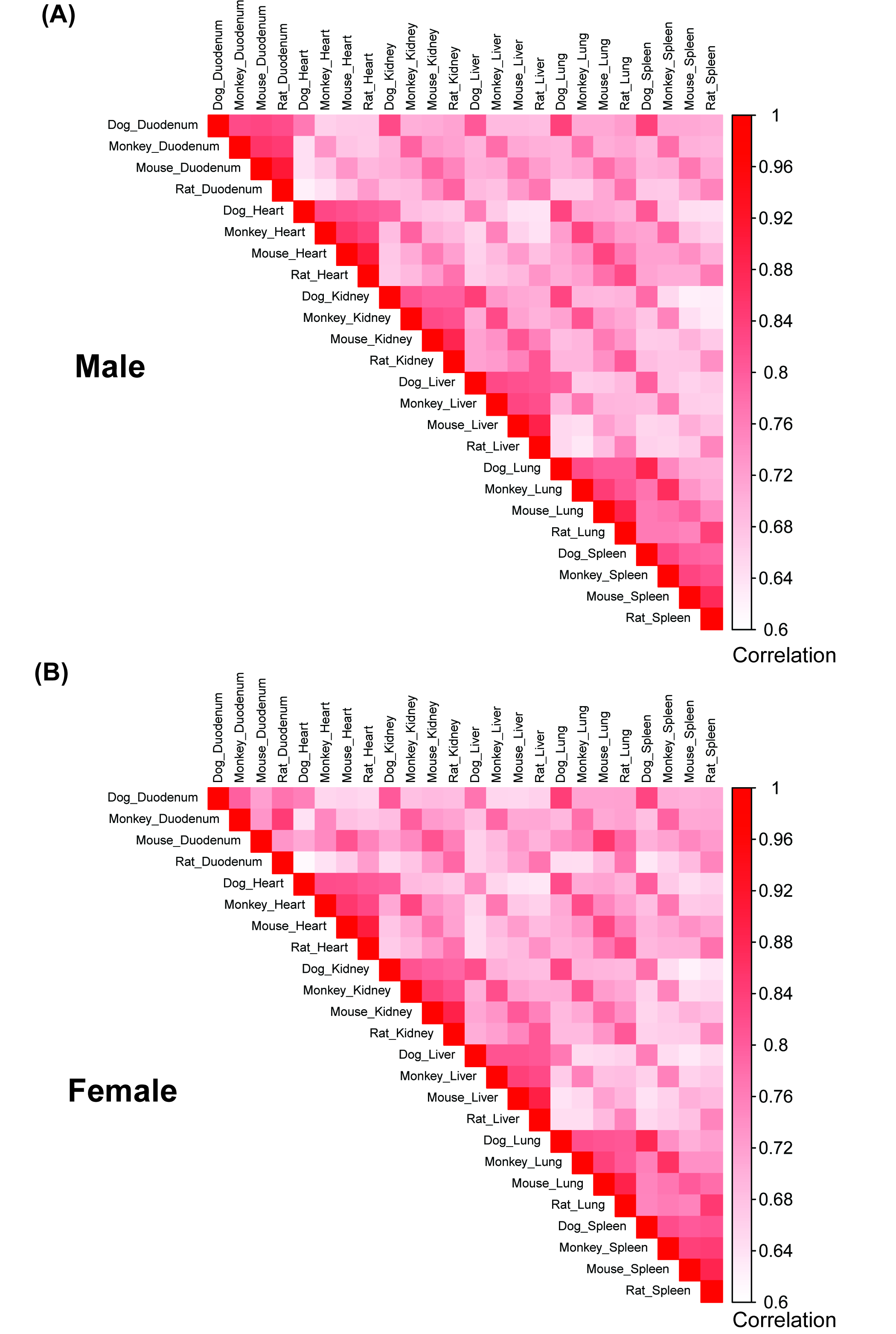

Supplement: Supplementary file 11 [file Image5.TIF]
