# Supplementary material for: Preclinical species gene expression database: Development and meta-analysis
Source: Front Genet. 2023 Jan 17;13:1078050. doi: 10.3389/fgene.2022.1078050 (PMC9887474; doi:10.3389/fgene.2022.1078050)
Supplement: Supplementary file 4 [file Table1.docx]

**Supplementary Table 1. List of Tissue Samples Collected for Histology**

| **Species** | **Tissue** |
| --- | --- |
| Rat | Liver, Kidney, and Heart |
| Mouse | Liver and Kidney |
| Dog | Liver, Kidney, Heart, and Lung |
| Monkey | All tissues |
